# GABAergic neuronal dysfunction underlies tremor in spinocerebellar ataxia 3

**DOI:** 10.1242/dmm.052329

**Published:** 2025-10-29

**Authors:** Animesh Banerjee, Moumita Chatterjee, Kah Junn Tan, Shermaine Tay, Kaibo Duan, Anand Kumar Andiappan, Shanshan Wu Howland, Yoshinori Aso, Sherry Shiying Aw

**Affiliations:** ^1^ Institute of Molecular and Cell Biology (IMCB), Agency for Science, Technology and Research (A*STAR), 61 Biopolis Drive, Singapore 138673; ^2^ Centre for Biomedical Informatics, Lee Kong Chian School of Medicine, Nanyang Technological University, 50 Nanyang Avenue, Singapore 639798; ^3^ Singapore Immunology Network (SIgN), Agency for Science, Technology and Research (A*STAR), 8A Biomedical Grove, Immunos, Singapore 138648; ^4^ A*STAR Skin Research Labs (A*SRL), Agency for Science, Technology and Research (A*STAR), 8A Biomedical Grove, Immunos, Singapore 138648; ^5^ Janelia Research Campus, Howard Hughes Medical Institute, Ashburn, VA 20147, USA

**Keywords:** *Drosophila*, Neurodegeneration, Tremor, VNC, SCA3, Spinocerebellar ataxia

## Abstract

Tremor is a common movement disorder associated with several neurodegenerative diseases, yet its mechanisms are not well understood. Using a machine-learning method, Feature Learning-based Leg segmentation and Tracking (FLLIT), we previously characterised gait and tremor signatures in a *Drosophila* model for spinocerebellar ataxia 3 (SCA3) and found them to be analogous to those in human SCA3. Here, we carried out a functional screen for neuronal populations that underlie tremor and found that dysfunction of a specific population of neurons in the ventral nerve cord (VNC) is necessary and sufficient for tremor. Adult-onset expression of mutant ATXN3 in, or genetic hypo-activation of, these neurons led to tremor, indicating their important role in adult motor control. RNA-sequencing and functional experiments showed that dysfunction of GABAergic neurons, and not that of other neurotransmitter populations tested, causes tremor. Finally, we identified a small subset of ∼30 predominantly GABAergic neurons within the adult VNC that are essential for smooth walking. This study demonstrates that tremor in SCA3 flies arises from GABAergic dysfunction, and that FLLIT can be used to dissect motor control mechanisms.

## Teaser

**Summary:** In a *Drosophila* model of spinocerebellar ataxia 3, tremor primarily arises from GABAergic dysfunction within the adult ventral nerve cord, including a subset of ~30 neurons for which impairment induces tremor.

## INTRODUCTION

Smooth, coordinated motor movements require proper functioning of the central and peripheral nervous systems, and the musculoskeletal system. Hence, neurodegenerative pathologies can manifest as movement abnormalities. One prevalent movement disorder is tremor – uncontrolled shaking of the body or appendages, which affects more than 1 in 20 people aged above 65 (Louis and McCreary, 2021). Tremor is associated with several diseases, including spinocerebellar ataxia 3 (SCA3), and can cause difficulty in performing basic tasks required for daily living. Treatments for tremor are lacking; current drugs available reduce tremor by an average of only 50% (Schneider and Deuschl, 2014), while surgical interventions, including deep brain stimulation, are highly invasive (Schneider and Deuschl, 2014).

The underlying pathophysiological causes of tremor are not well understood (Kuo et al., 2019). Although human brain anatomical regions have been implicated – including cerebellar circuits, the red nucleus, inferior olivary nucleus and the dentate nucleus (Miskin and Carvalho, 2018) – detailed pathway mechanisms remain poorly characterised. There are several hypotheses for how tremor is generated. Some have posited a mechanism of aberrant positional correction in response to proprioceptive feedback due to malfunctioning circuits (Fink et al., 2014). Another hypothesis is that tremor results from underlying neural oscillators with spontaneous rhythmicity (McAuley and Marsden, 2000). Yet others have posited a biophysical underpinning based on natural mechanical resonant frequencies of the extremities (Joyce and Rack, 1974). Understanding tremor is further complicated by its substantial phenotypic heterogeneity, with high individual variance (Szewczyk-Krolikowski et al., 2014; van Rooden et al., 2011; Wolters, 2008). Hence, our understanding of tremor, in order to better manage it, can benefit from its study in a genetically controlled model system. The fruit fly *Drosophila* has a million-fold fewer brain cells than humans, yet shares 60% of our genes. Despite the differences in anatomy and scale between human and fly brains, *Drosophila* models have been instrumental in elucidating fundamental mechanisms of normal brain function and human neurological disorders (Zoghbi and Warren, 2010; McGurk et al., 2015). Genomic integration of wild-type or variant forms of human disease-associated genes, such as mutant ataxin-3 [*ATXN3*; the gene mutated in Machado–Joseph disease, also known as SCA3 (Warrick et al., 1998)], into the *Drosophila* genome has enabled investigation of disease pathophysiology. In addition, fly models have a large arsenal of genetic and molecular tools for targeted neuronal manipulation and visualisation.

Even though gait and tremor variations form the basis of disease classification in human patients (Fahn, 2011), and methods have been developed to quantify gait in flies (Mendes et al., 2013; Wu et al., 2019), there is a lack of high-resolution characterisation of movement defects, such as tremor, in animal models of neurodegeneration, which could help to link molecular defects to circuit perturbation and resultant behavioural movement deficits. To enable the measurement of specific movement disorders in the fly model, we previously developed an automated, machine learning-based leg tracking method, Feature Learning-based Leg segmentation and Tracking (FLLIT; Wu et al., 2019), which segments limbs and tracks limb tip positions of freely walking flies captured on high-speed video (Wu et al., 2019). Using FLLIT, we characterised high-resolution gait and tremor features in *Drosophila* neurodegeneration models of SCA3 and Parkinson's disease (PD) for the first time. SCA3 is the most common of the family of neurodegenerative diseases known as spinocerebellar ataxias and is caused by an expansion of the polyglutamine (polyQ) repeat region, encoded by predominantly CAG trinucleotide repeats in *ATXN3* (McLoughlin et al., 2020). ATXN3 has multiple cellular roles, including in the ubiquitin-proteasome pathway (Chai et al., 2004; Burnett et al., 2003; Schmitt et al., 2007) and in transcriptional regulation (Evert et al., 2006). Aggregation of mutant ATXN3 (mutATXN3; with an expanded polyQ repeat region) and alpha-synuclein are key hallmarks of SCA3 (McLoughlin et al., 2020) and PD (Calabresi et al., 2023), respectively. We examined gait in well-established fly models of SCA3 and PD, in which a mutant form of ATXN3 with 84 expanded polyglutamine repeats [mutATXN3-(CAG)_84_] and alpha-synuclein (Chouhan et al., 2016; Feany and Bender, 2000), respectively, were expressed, both of which have no apparent homologue in the fly. Analysis of these flies with FLLIT showed that pan-neuronal expression (with Elav-Gal4) of mutATXN3-(CAG)_84_, but not the wild-type form of ATXN3 with 27 expanded polyglutamine repeats [wtATXN3-(CAG)_27_] (Warrick et al., 1998), led to an ataxic, lurching and stumbling gait (Wu et al., 2019). In contrast, pan-neuronal expression of alpha-synuclein (Chouhan et al., 2016; Feany and Bender, 2000), the elevated expression of which is associated with PD (Ibáñez et al., 2004), caused a rigid gait. We also observed that the fly model of SCA3 (Warrick et al., 1998) exhibited action tremor when walking (Wu et al., 2019). These gait and tremor features resemble that of the respective human diseases, and establish the SCA3 fly as a suitable model with which to study action tremor. Here, we built on this observation to identify and characterise neurons that are involved in the tremor phenotype.

First, through Gal4 and Split-Gal4-based genetic screening, we identified specific neuronal populations in the fly ventral nerve cord (VNC) for which dysfunction is necessary and sufficient for generation of tremor. Second, using a heat shock-inducible genetic system, we demonstrated that activation of tremor-related neurons in the adult stage is sufficient to induce tremor. Third, we found that depolarisation and ablation of this population of neurons generated tremor, suggesting that tremor generated by mutATXN3-(CAG)_84_ appears to derive from the resultant dysfunction of the neurons and not specific molecular perturbations caused by mutATXN3-(CAG)_84_ protein itself. Finally, we identified a small subset of ∼30 predominantly GABAergic neurons within the VNC for which loss of function is sufficient to drive leg tremors during walking. Together, our results underscore how dysfunction of a highly specific subset of neurons leads to tremor. To our knowledge, this is the first study to broadly screen neuronal populations for tremor phenotypes in any animal model. These results also show that FLLIT is a robust platform for study of tremor mechanisms and to dissect the neurocircuitry underlying tremor.

## RESULTS

### Identification of neuronal populations for which dysfunction leads to tremor in flies

**Fig. 1.**
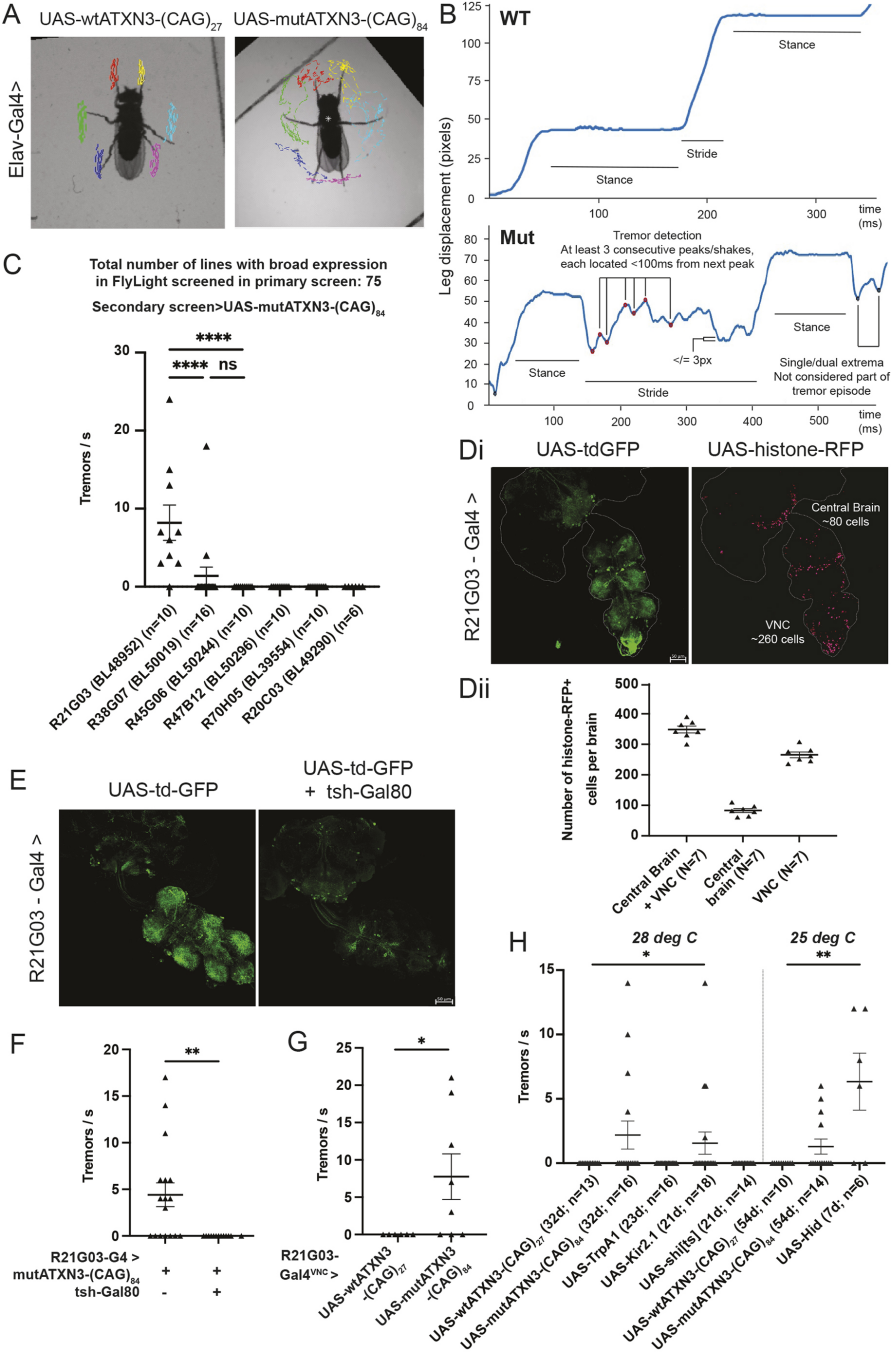
**Dysfunction of a population of fly ventral nerve cord (VNC) neurons leads to tremor.** (A) Video stills (from Movies 1 and 2) of leg-tracked flies with pan-neuronal expression of wtATXN3-(CAG)_27_ and mutATXN3-(CAG)_84_. (B) Representative Feature Learning-based Leg segmentation and Tracking (FLLIT)-tracked leg traces from wtATXN3-(CAG)_27_ (top) and mutATXN3-(CAG)_84_ (bottom) flies, demonstrating the parameters used to determine tremor events. (C) A genetic screen for drivers that cause tremor when used to express mutATXN3-(CAG)_84_; shown here is the secondary screen of top candidates (R21G03, *n*=10; R38G07, *n*=16; R45G06, *n*=10; R47B12, *n*=10, R70H05, *n*=10; R20C03, *n*=6) from the primary screen (the full dataset is in Table S1). (Di) R21G03-Gal4 is expressed in the adult central brain and VNC. Scale bar: 50 µm. (Dii) R21G03-Gal4 is expressed in ∼90 central brain cells and ∼260 VNC cells (*n*=7). (E) tsh-Gal80 represses R21G03-Gal4 expression in the VNC. Scale bar: 50 µm. (F) Tremor is abolished in wtATXN3-(CAG)_84_ flies with tsh-Gal80-induced repression of R21G03-Gal4 expression in the VNC (*n*=13) compared to those without tsh-Gal80 (*n*=17). (G) Tremor is induced in flies with expression of mutATXN3-(CAG)_84_ only in the VNC (*n*=8) compared to those with expression of mutATXN3-(CAG)_27_ in the same domain (*n*=6). (H) Functional manipulation of R21G03-Gal4 neurons suggests that either cell death (UAS-Hid, *n*=6) or preventing depolarisation (UAS-Kir2.1, *n*=18) of these neurons can cause tremor. d, days. All sample sizes shown in figure for brevity. All experiments were carried out at least twice. Tremor analyses were carried out on single fly biological replicates. All statistics were carried out with non-parametric Mann–Whitney (when comparing two samples) and one-way ANOVA with Dunn's multiple correction (when comparing more than two samples). Data are means±s.e.m. *P<0.05; **P<0.01; ****P<0.0001.

We used FLLIT (Wu et al., 2019) to segment and track leg claw positions of walking flies from high-speed video (Fig. 1A; Movies 1 and 2). We found that although the leg paths of flies expressing pan-neuronal wild-type ATXN3 [wtATXN3-(CAG)_27_] were relatively smooth (Fig. 1B; Movie 1), the leg paths traced by flies expressing pan-neuronal mutATXN3 [mutAXTN3-(CAG)_84_] during a stride event were irregular and ‘shaky’ (Fig. 1B; Movie 2). Because tremor is defined as an involuntary, oscillatory movement of a body part (Bhatia et al., 2018), we quantified the number of shaking events of each leg using the FLLIT-tracked leg traces during walking. We used parameters that were established in our previous study, which defined a tremor episode as comprising at least three consecutive leg shakes occurring within 100 ms (Wu et al., 2019), about twice the average period of a stride in wild-type flies (Fig. 1B; see Materials and Methods for more details).

Because pan-neuronal expression of mutATXN3-(CAG)_84_ led to strong tremor, but not overexpression of alpha-synuclein (Wu et al., 2019), we wondered whether other disease-associated expanded polyglutamine tract transgenes could cause tremor. We found that pan-neuronal expression of UAS-poly-CAG (a peptide consisting of 63 polyglutamine repeats) (Kazemi-Esfarjani and Benzer, 2002), UAS-mutant-ATXN1 [expanded polyglutamine repeat form of ataxin-1 (*ATXN1*), the causative gene for spinocerebellar ataxia 1] (Fernandez-Funez et al., 2000) and UAS-mutant-HTT [mutant expanded polyglutamine repeat form of huntingtin (*HTT*), the causative gene for Huntington's disease] (Barbaro et al., 2015) did not result in tremor (Fig. S1A). To investigate the mechanisms that underlie mutATXN3-(CAG)_84_-induced tremor, we carried out a genetic screen to narrow down tremor-relevant populations by expressing mutATXN3-(CAG)_84_ in smaller subsets of neurons using enhancer-based Gal4 drivers (Pfeiffer et al., 2008, 2010; Dionne et al., 2017; Tirian and Dickson, 2017 preprint). Although misexpression studies can sometimes be difficult to interpret, in this case we were not looking for endogenous function of ATXN3 (which is not conserved in *Drosophila*) but rather using mutATXN3-(CAG)_84_ as a reporter of neuronal populations for which normal function is required for smooth motor movements, in light of knowing that its pan-neuronal expression causes tremor (Wu et al., 2019).

We screened 75 driver lines, chosen for their relatively wide expression (FlyLight) (Jenett et al., 2012) (Table S1A,B), in two stages. Because pan-neuronal expression of mutATXN3-(CAG)_84_ with Elav-Gal4 showed a phenotype with ∼80% penetrance and an effect size of 1.38 (Cohen's *d*) versus Elav-Gal4>wtATXN3-(CAG)_27_ (Wu et al., 2019), we carried out the screen with a relatively small sample size per driver line (*n*=3). A line with 80% penetrance would have a 99% probability of showing tremor in at least one of three flies tested (i.e. the chance that none of the flies would show tremor is ∼0.2^3^). We previously found that poorly climbing Elav-Gal4>wtATXN3-(CAG)_84_ flies tended to show more severe gait defects (Wu et al., 2019). To see whether this was also true for tremor, we analysed both tremor and climbing score (height climbed) in these flies and found a moderate negative correlation; flies that were poor climbers tended to show more tremor, although good climbers could also exhibit tremor [correlation coefficient (*r*)=−0.44; *P*=−0.0516)] (Fig. S1B). Hence, to select flies for recording, single fly climbing assays were carried out at 50±1 days, and the three poorest climbers were selected for analysis. To reduce false negatives due to the small sample size, we initially lowered the criteria for a tremor to two shakes per tremor [from three shakes per tremor (Wu et al., 2019)]. From the first primary screen of 30 lines (Table S1A), we selected the top candidate lines and carried out a secondary screen with larger sample sizes and a stricter criterion of three shakes/s defined as a tremor. Only R21G03-Gal4 showed a statistically significant rate of tremor when used to express mutATXN3-(CAG)_84_ [Fig. 1C; see Movies 3 and 4 for R21G03-Gal4-driven wtATXN3-(CAG)_27_ and mutATXN3-(CAG)_84_, respectively]. Because we observed false positives with the initial two shakes/s primary screen, and the final candidate line was the best performer in the primary screen (Fig. 1C), we tightened the stringency to three shakes/s for a second primary screen of another 45 other driver lines selected from FlyLight (Jenett et al., 2012). None of these lines showed tremor (Table S1B), and we used this stricter criterion going forward. Consistent with our previous study (Wu et al., 2019), we observed incomplete penetrance of the tremor phenotype in flies (see Discussion). With the exception of R21G03-Gal4 (of which ∼80% of flies showed tremor; Fig. 1C), two out of the remaining 167 flies screened showed tremor, a background rate of ∼1.2%. Hence, of a total of 75 lines screened that are broadly expressed in the adult fly nervous system, only R21G03-Gal4 exhibited tremor, and we selected it for further analysis.

R21G03-Gal4 is expressed in both the central brain and VNC (Fig. 1Di). A count of the number of cells using UAS-Histone-RFP showed expression in ∼80 cells in the central brain and ∼260 cells in the VNC (Fig. 1Dii). Reporter-only controls showed almost no expression (Fig. S1C). Blocking R21G03-Gal4 expression in the VNC using tsh-Gal80 (Simpson, 2016) diminished expression of UAS-tdGFP and mutATXN3-(CAG)_84_ expressed from this driver in the VNC (Fig. 1E; Fig. S1D) and abolished tremor caused by mutATXN3-(CAG)_84_ (Fig. 1F), demonstrating that its expression in the VNC is necessary for tremor. We next asked whether R21G03-Gal4-driven mutATXN3-(CAG)_84_ expression in the VNC is sufficient for tremor using tsh-LexA, LexAOp>FLP to selectively remove Tubulin>Gal80 repression only in the VNC [tsh-Gal4 has minimal central brain expression (Simpson, 2016)]. This was sufficient to induce tremor (Fig. 1G). Therefore, expression of mutATXN3-(CAG)_84_ in a population of ∼260 VNC neurons is necessary and sufficient to cause tremor in the fly model of SCA3.

We next employed genetic tools to understand the mechanisms that underlie the observed tremor phenotype. We used R21G03-Gal4 to specifically depolarise (UAS-TrpA1) (Pulver et al., 2009), prevent depolarisation of (UAS-Kir2.1) (Baines et al., 2001), block chemical neuronal transmission of (UAS-*shibire^ts^*) (Kitamoto, 2001) or ablate (UAS-Hid) (Zhou et al., 1997) these neurons (Fig. 1H). We found that tremor was phenocopied by neuronal inhibition with UAS-Kir2.1 (*P*<0.05; *n*=18) and ablation with UAS-Hid (*P*<0.01; *n*=6), but not by neuronal activation with UAS-TrpA1 (Fig. 1H), suggesting that the tremor phenotype is induced by neuronal hypoactivation. Curiously, loss of neuronal chemical transmission of R21G03-Gal4 neurons using UAS-*shibire^ts^* did not cause tremor. We ascertained that the UAS-*shibire^ts^* transgene was functional, as driving its expression in all neurons with Elav-Gal4 was lethal (data not shown). Although UAS-*shibire^ts^* (Kitamoto, 2001) and UAS-TrpA1 (Pulver et al., 2009) are usually analysed at above 30°C, we carried out our experiments at 28°C, as they required chronic expression of these transgenes, and incubation of flies at 30°C led to premature death within a few days. Hence, the lack of effect could arise from inefficient expression of UAS-*shibire^ts^* at 28°C.

With this in mind, the difference between UAS-Kir2.1 and UAS-*shibire^ts^* (Kitamoto, 2001) nonetheless led us to wonder whether tremor generation can result from aberrant electrical neurotransmission, perhaps due to dysregulation of gap junction activity. Because there are conflicting data in the field regarding the role of gap junctions in essential tremor (ET) (Loewenstein, 2002; Long et al., 2002; Martin and Handforth, 2006), we asked whether downregulation of innexins [subunits of gap junctions in insects (Phelan, 2005)] in R21G03-Gal4-expressing neurons could cause tremor. However, RNA interference (RNAi)-mediated downregulation of all eight different innexins, either singly or in pairs [as innexins can form heteromers (Lehmann et al., 2006)], under R21G03-Gal4 failed to elicit tremor (Fig. S1E). Although it is important to note that we did not validate innexin knockdown by reverse transcription quantitative PCR to confirm that these negative results hold despite strong innexin knockdown, there does not appear to be a substantial role for innexins in tremor induction. Thus, although blocking chemical transmission via UAS-*shibire^ts^* (Kitamoto, 2001) within the parameters of our experiment did not cause tremor, hypoactivation of R21G03-Gal neurons with UAS-Kir2.1 and UAS-Hid led to robust tremor, demonstrating that loss of function of these neurons is sufficient to cause tremor.

### Expression of mutATXN3-(CAG)_84_ in adult flies is sufficient for tremor induction

To better understand the mechanism of tremor generation, we wanted to further narrow down the population of tremor-relevant neurons. We utilised a genetic intersectional method (Split-Gal4) to express mutATXN3-(CAG)_84_ in smaller subsets of neurons within R21G03-Gal4. The Split-Gal4 system uses enhancer promoters to drive two halves of Gal4 protein, the activation domain (AD) and DNA-binding domain (DBD). The subset of cells that lie at the intersection of both drivers will express both halves and, hence, assemble functional Gal4 protein (Dionne et al., 2017; Tirian and Dickson, 2017 preprint).

**Fig. 2.**
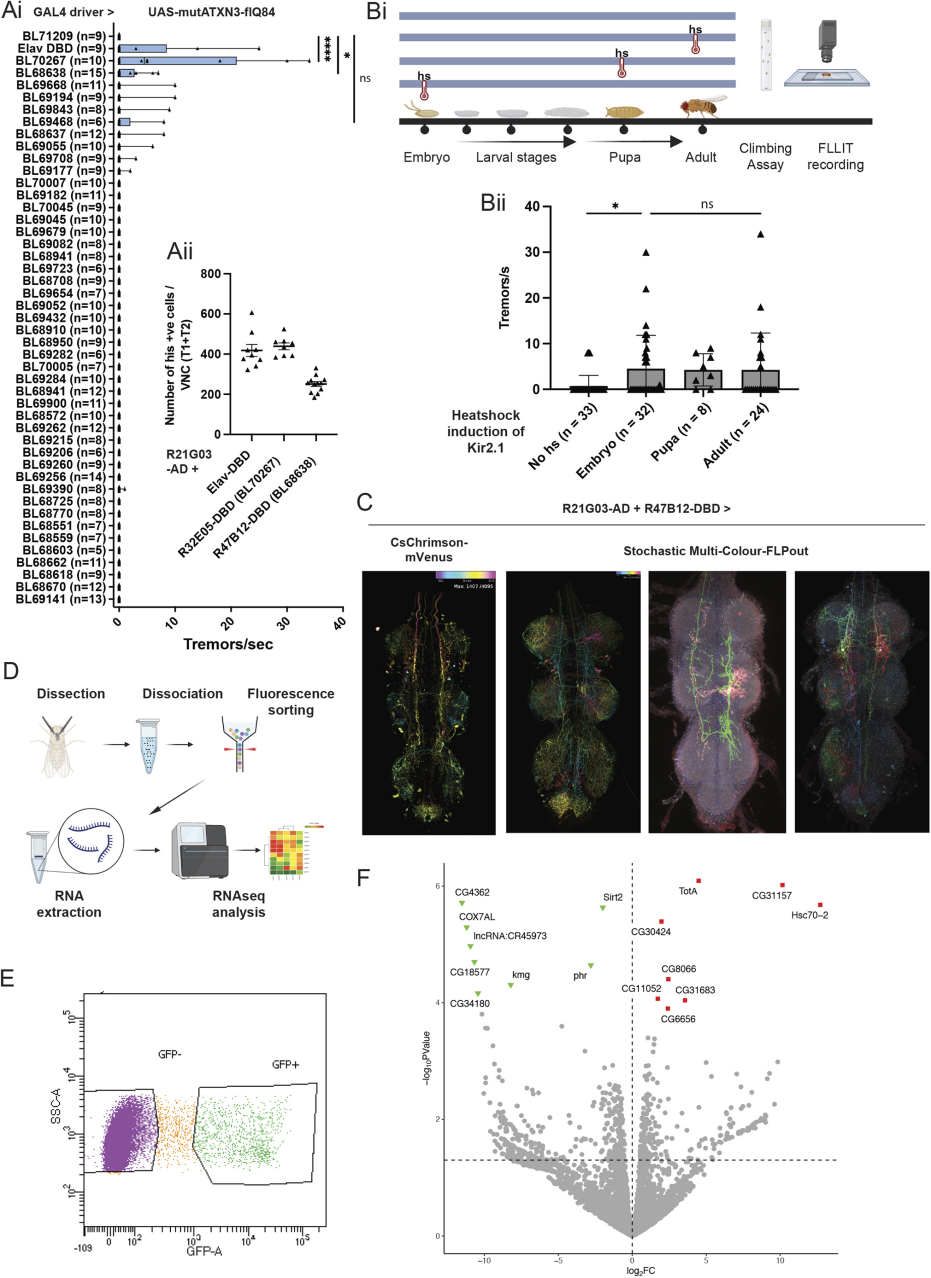
**Functional and RNA-sequencing analysis of a narrowed subset of tremor-associated neurons in the adult VNC.** (Ai) A Split-Gal4 screen combining R21G03-AD and mutATXN3-(CAG)_84_ with a panel of Gal4-DBD lines led to identification of two Split-Gal4 combinations that cause tremor. All screen sample sizes shown in figure for brevity. (Aii) Number of VNC neurons (T1, first thoracic segment; T2, second thoracic segment of VNC) expressed in the intersection of R21G03-AD and Elav-DBD (*n*=9), R32E05-DBD (*n*=8) or R47B12-DBD (*n*=13). (Bi) Schematic showing heat shock (hs) of flies at various developmental time points, followed by climbing assay and FLLIT recording. (Bii) Activation of Kir2.1 in R21G03-AD+R47B12-DBD neurons using hsFLP in adults (*n*=24) causes tremor at the same rate as in embryos (*n*=32) (no hs, *n*=33; pupal heat shock, *n*=8). (C) Expression of R21G03-AD and R47B12-DBD from FlyLight. For the left-most panel showing the combined split drivers driving CsChrimson-Venus, the pixel colour encodes depth position (depth-based colour gradient), where the ventral-most section is in yellow, and subsequent sections are coded according to the key in the top-left corner of the figure panel. In the stochastic multi-colour FLPout images, each colour is a stochastically labelled single neuron. (D) Schematic of RNA-sequencing experiment with fluorescence-activated cell sorting (FACS)-sorted GFP-expressing neurons. (E) FACS of dissociated R21G03-Gal>GFP brains allowed isolation of both the neurons of interest (GFP^+^) and the non-labelled neurons (GFP^−^) for subsequent transcriptional profiling. (F) Volcano plot for all genes detectable in the wild-type versus mutant ATXN3 dataset. Differentially expressed [false discovery rate (FDR)-corrected *P*<0.05] genes are highlighted in blue for genes that were upregulated when mutant ATXN3-(CAG)_84_ was expressed compared to wild-type ATXN3-(CAG)_27_, and in red for genes that were downregulated. Horizontal and vertical dashed lines correspond to FDR-corrected *P*<0.05 and log_2_FC=0, respectively. The tremor experiments were repeated at least twice. Tremor analyses were carried out on single fly biological replicates. All statistics were done with one-way ANOVA with Dunn's multiple correction. Data are means±s.e.m. ns, not significant. *P<0.05; ****P<0.0001.

We selected 164 DBD lines from FlyLight (Jenett et al., 2012) that are expressed in pro- and meta-thoracic regions of the VNC (in which the primary population of interest resides; Fig. 1E,F). We first asked which of these lines intersect with R21G03 expression by combining each DBD line with R21G03-AD and a UAS-membrane-GFP reporter. Fifty-three of the 164 DBD lines drove visible GFP expression in the VNC, demonstrating intersection with R21G03-AD (Table S2). We noted that the control/Empty-DBD line (BL71209) drove GFP expression in neuroblast-like cells in the central brain when crossed to R21G03-AD (Fig. S2A). Therefore, for the functional tremor screen, we chose lines that overlapped with R21G03-AD in the VNC, as well as a few other non-intersecting negative control lines, but did not further examine lines for which expression resembled that of the Empty-DBD line (Table S2). We screened these DBD Split-Gal4 lines in combination with R21G03-AD and UAS-mutATXN3-(CAG)_84_. Of the 428 flies screened from these 53 DBD lines, which intersect with the R21G03 population that already showed tremor, 20 flies exhibited tremor. More than half were from two lines that exhibited statistically significant tremor rates: R32E05-DBD (BL70267) (60% with tremor, *P*<0.0001; *n*=10) and R47B12-DBD (BL68638) (33% with tremor, *P*<0.05; *n*=15) (Fig. 2Ai). Therefore, the background rate of tremor in the rest of the lines was 9/403 flies, or ∼2.2%.

A count of the neurons expressed in the intersection in the first and second thoracic segments of the VNC revealed that although R32E05-DBD showed high tremor rates, the number of neurons that intersected between that line and R21G03-AD was still a large population, comparable to Elav-DBD, which is expressed in the theoretical limit of the expression pattern of R21G03 (Fig. 2Aii; Fig. S2B). R47B12-DBD narrowed down the subset of tremor-relevant neurons almost by half compared with Elav-DBD (Fig. 2Aii; Fig. S2B). We noted that the number of VNC cells expressing R21G03-Gal4 (∼260; Fig. 1D) was less than that for R21G03-AD+Elav-DBD (∼400; Fig. 2Aii), suggesting that the split combination drives more strongly, or in a larger number of cells, than the single Gal4 driver.

Although we selected the Gal4 and Split-Gal4 lines based on their adult expression patterns from FlyLight, these lines would drive mutant SCA3 in flies starting from the embryonic stage (or at the timepoint at which the drivers turn on during development). Hence, we wanted to determine the critical time period for induction of SCA3-associated tremor. Because expression of Kir2.1 with R21G03-Gal4 led to tremor (Fig. 1G), we made use of a heat-shock (hs)-inducible Kir2.1 transgene (von Philipsborn et al., 2011), which allowed us to inactivate tremor neurons starting at various time points (Fig. 2Bi). We induced Kir2.1 expression in R21G03-AD+R47B12-DBD neurons (Fig. 2Bii) at embryonic, pupal or adult stages [we also tested the effect of no-heat shock (no hs), which is expected to prevent Kir2.1 induction, as a negative control]. The average number of tremors induced per second when Kir2.1 was turned on at the embryo stage was the same as when it was induced at the adult stage (Fig. 2Bii). Hence, expression of mutATXN3 in adult flies is sufficient for tremor induction, suggesting that developmental perturbation of R47B12-DBD neurons does not substantially contribute to tremor generation.

### Transcriptional profiling of tremor-associated neurons

Guided by stochastic multi-colour FLPout data (FlyLight) (Shuai et al., 2023) showing R21-G03-AD and R47B12-DBD neurons projecting longitudinally along the VNC (Fig. 2C), and supported by our tsh-Gal80 and Kir2.1 experiments highlighting the critical role of adult VNC neurons in tremor generation, we next sought to gain molecular insight into the identity of these neurons by performing RNA sequencing on driver neuronal populations isolated from the adult VNC. We chose the R21G03-Gal4 driver, which causes a high rate of tremor when used to drive mutATXN3-(CAG)_84_ (Fig. 1C). We labelled (R21G03-Gal4>UAS-CD8-GFP) and used fluorescence-activated cell sorting (FACS) of dissociated *Drosophila* VNC to isolate both GFP^+^ (*n*=3; where each sample consisted of ∼500 cells) and GFP^−^ (*n*=4) neurons in the wild-type state following established procedures (Croset et al., 2018) (Fig. 2D,E). Because there were only several hundred neurons expressing this driver (Fig. 1D), making their isolation challenging, we simultaneously carried out a technical control experiment, in which all neurons were labelled [neuronal Synaptobrevin (nSyb)-Gal4>UAS-CD8-GFP]. Labelled (GFP^+^) (*n*=3) and non-labelled (GFP^−^) (*n*=3) neurons were also isolated from this cross, and RNA was extracted from all samples for subsequent bulk RNA sequencing by direct lysis of a limited input of 500 cells per sample.

Our technical control experiment showed robust neuronal signatures in the nSyb-Gal4>GFP^+^ population, with upregulation of neuronal genes like *nSyb* itself [log fold change (logFC)>12.7; false discovery rate (FDR) *P*<0.05], *Synaptotagmin* 4 (*Syt4*) (logFC>16.4; FDR *P*<0.05) and *Down syndrome cell adhesion molecule 2* (*Dscam2*) (logFC>12.5; FDR *P* value<0.002), while glial-associated genes like *reversed polarity* (*repo*) (logFC>13.5; FDR *P*<0.05) and *Excitatory amino acid transporter 1* (*Eaat1*) (logFC>14.9; FDR *P*<0.05) were upregulated in the GFP^−^ (glial) population (Fig. S2C, Table S3). Principal component analyses (PCAs) showed that the global transcriptome of the two populations segregated away from each other, with the three neuron replicates forming a tight cluster (Fig. S2D). These results demonstrate effective separation and transcriptional profiling of GFP-labelled cells.

We then examined the transcriptional profile of R21G03-Gal4>UAS-CD8-GFP cells. We found that the *Glutamic acid decarboxylase 1* (*Gad1*) transcript was upregulated in GFP^+^ compared to GFP^−^ cells, with the eighth lowest uncorrected *P*-value (*P*<0.0005) of the 8115 transcripts in this comparison; however, none of the genes in the dataset, including *Gad1*, were statistically different after FDR correction (Table S4). In this comparison, GFP^−^ cells also included glia; however, neurotransmitter markers were not universally upregulated in GFP^+^ cells [e.g. *Choline acetyltransferase* (*ChAT*) was downregulated with logFC ∼−1.42 (FDR ns)]. When comparing R21G03-Gal4>UAS-CD8-GFP to nSyb-Gal4>UAS-CD8-GFP neurons, we also observed that *Gad1* was upregulated, with the 14th lowest uncorrected *P*-value (logFC ∼1.2; uncorrected *P*=∼0.001), although the FDR was again not statistically significant (Table S5). These analyses suggested that R21G03-expressing neurons comprise a population of GABAergic neurons; however, the lack of statistical significance suggested that not all the R21G03-Gal4 neurons are Gad1 expressing, and/or [more likely, because ∼38% of, or ∼10,000, VNC neurons are GABAergic (Allen et al., 2020)], that a large percentage of non-R21-G03 neurons also express Gad1.

To examine potential molecular mechanisms underlying tremor induction by mutATXN3, we expressed wtATXN3-(CAG)_27_ (*n*=3) or mutATXN3-(CAG)_84_ (*n*=5) in R21G03-Gal4 neurons labelled with GFP, sorted for GFP^+^ VNC cells, and carried out RNA sequencing to characterise differences in gene expression between the two groups. We carried out this experiment in young flies, as neurodegeneration resulting from mutATXN3 expression has been previously reported (Warrick et al., 1998), and we observed pronounced loss of mutATXN3-(CAG)_84_-expressing neurons in aged flies (Fig. S2E). Interestingly, when R21G03-Gal4 was used to simultaneously drive both mutATXN3-(CAG)_84_ and tdGFP, mutATXN3 aggregates formed in some GFP-expressing cells but not others (Fig. S2Fi). We used a nuclear marker, Histone-RFP, to examine colocalisation with nuclear aggregates of mutATXN3, and found that although some cells that expressed Histone-RFP (pseudo-coloured in green in Fig. S2Fii) also exhibited mutATXN3 aggregates (in pink; white arrows in Fig. S2Fii), some cells that expressed Histone-RFP did not exhibit mutATXN3 aggregation (orange arrows in Fig. S2Fii). This suggests that there is cellular heterogeneity in the propensity to aggregate mutATXN3 within this population, which is interesting to consider together with the behavioural heterogeneity and incomplete penetrance of the tremor phenotype. In light of this, to isolate transcriptomic changes due to mutATXN3-(CAG)_84_ expression and minimise the effect of secondary transcriptomic changes arising from cell death, we selected young (7- to 9-day-old) flies for RNA-sequencing analysis. PCAs of the global gene expression profiles showed clear separation between the wild-type and mutant samples, indicative of different expression profiles (Fig. S2G). Interestingly, expression of mutATXN3-(CAG)_84_ in R21G03 neurons appeared to result in stronger clustering of the transcriptome of R21G03 neurons than expression of wtATXN3-(CAG)_27_ (Fig. S2G), suggesting that mutATXN3 had a strong effect on changing the gene expression of these neurons, to result in altered expression of a common set of genes. There were 21 genes that were significantly different (*P*<0.05) after FDR correction (Fig. 2F; Table S6). Some of these genes, such as the protein-folding chaperone *Heat shock protein 70 cognate 2* (*Hsc70-2*) and the deacetylase *Sirtuin 2* (*Sirt2*), have previously been implicated in polyglutamine diseases (Kuo et al., 2013; Luthi-Carter et al., 2010). Other heat-shock proteins have also been shown to be downregulated when mutATXN3 is overexpressed (Wen et al., 2003). However, most of the differentially expressed genes have not been characterised or have unknown function.

To analyse the altered gene set, we performed a standard Gene Ontology (GO) analysis (Fig. S2H-J). The analyses revealed that the top biological processes affected were in regulation of calcium (*P*<0.0001), amine (*P*<0.001) and ion transport (*P*<0.001). A disproportionate number of transcripts coding for pre-synaptic active zone proteins were dysregulated (*P*<0.001), and genes for which molecular functions relate to G protein-coupled receptor (GPCR) binding were also significantly affected (*P*<0.001). GPCR and calcium binding, transport of calcium ions, axonogenesis and the chaperone complex have previously been implicated in mouse models of SCA3 or in patients with ataxia (Eidhof et al., 2019; McLoughlin et al., 2020; Zeng et al., 2018).

### GABAergic neuron dysfunction underlies tremor in the fly model of SCA3

**Fig. 3.**
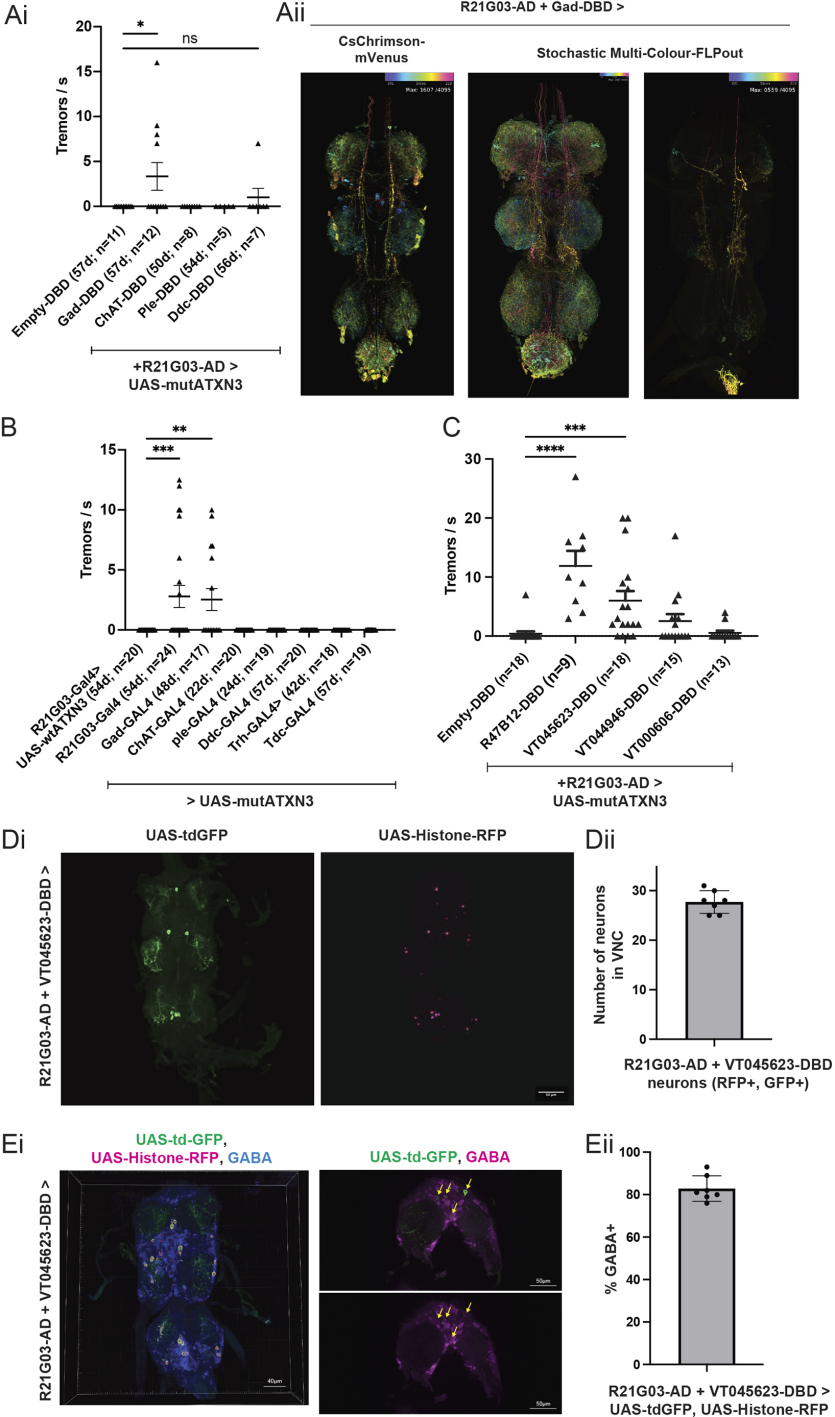
**GABAergic neurons underlie spinocerebellar ataxia 3-associated tremor.** (Ai) A Split-Gal4 screen for tremor combining R21G03-AD and mutATXN3-(CAG)_84_ with Gal4-DBD expressed under various neurotransmitter promoters (Empty-DBD, *n*=11; Gad-DBD, *n*=12; ChAT-DBD, *n*=8; Ple-DBD, *n*=5; Ddc-DBD, *n*=7). (Aii) R21G03-AD+Gad-DBD>CsChrimson-mVenus and R21G03-AD+Gad-DBD>Stochastic Multi-Colour-FLPout (Nern et al., 2015) [from FlyLight (Jenett et al., 2012)]. (B) Neurotransmitter Gal4 driver lines (R21G03-GAL4, *n*=24; Gad-GAL4, *n*=17, ChAT-GAL4, *n*=20; Ple-GAL4, *n*=19; Ddc-GAL4, *n*=20; Trh-GAL4, *n*=18; Tdc-GAL4, *n*=19) were used to drive mutATXN3-(CAG)_84_, and the resulting flies were assayed for tremor compared to R21G03-GAL4>UAS-wtATXN3-(CAG)_27_ (*n*=20). (C) Split-Gal4 combination of R21G03-AD+VT045623-DBD (63d, *n*=18) causes tremor when used to drive mutATXN3-(CAG)_84_, compared to Empty-DBD (*n*=18). Also tested: R47B12-DBD (*n*=9), VT044946-DBD (*n*=15) and VT000606-DBD (*n*=13). (Di) Confocal *z*-stack of R21G03-AD+VT45623-DBD driving UAS-tdGFP and UAS-Histone-RFP. Scale bar: 50 µm. (Dii) There are ∼25-30 neurons in the intersection of R21G03-AD+VT45623-DBD in adult VNC (*n*=7). (Ei) Left: Imaris colocalisation analysis of Histone-RFP driven by R21G03-AD+VT45623-DBD, with GABA immunostaining. Scale bars: 40 µm (left), 50 µm (right). Right: single section of R21G03-AD+VT45623-DBD>UAS-tdGFP co-stained with GABA. Yellow arrows indicate GABA-positive neurons. (Eii) 80% of the neurons in the intersection of R21G03-AD+VT45623-DBD are GABA positive (*n*=7). The experiments related to tremor measurement were carried out at least twice. Tremor analyses were carried out on single fly biological replicates. All statistics were done with one-way ANOVA with Dunn's multiple correction. Data are means±s.e.m. ns, not significant. *P<0.05; **P<0.01; ***P<0.001; ****P<0.0001.

The RNA-sequencing analyses hinted at increased expression of *Gad1*, a marker of GABAergic neurons, in R21G03 neurons; however, the result was not statistically significant after FDR correction. Hence, we independently investigated whether perturbation of GABAergic neurons and other neurotransmitter neurons in this population was sufficient to induce tremors. We intersected R21G03-AD with DBD lines expressed under various neurotransmitter drivers to drive mutATXN3-(CAG)_84_, and found that combination with Gad1-DBD (*P*<0.05; *n*=12), but not with ChAT, Ple or Ddc-DBD, led to tremor (Fig. 3Ai). The intersection of R21G03-AD and Gad1-DBD was extensively expressed in the VNC, and stochastic multi-colour FLPout data from FlyLight (Jenett et al., 2012) showed their projection along the VNC (Fig. 3Aii). The Gad1-DBD functional data demonstrate that at least part of the population of R21G03-Gal4 neurons that are required for smooth motor movements is GABAergic. This result led us to ask whether dysfunction of any other neurotransmitter neuron population across the nervous system could cause tremor. Of the six neurotransmitter Gal4 driver lines tested, only Gad1-Gal4 led to tremor when used to drive mutATXN3-(CAG)_84_ (*P*<0.01; *n*=17) (Fig. 3B). Therefore, from the Split-Gal4 and Gal4 experiments with neurotransmitter drivers, we conclude that GABAergic neuronal dysfunction underlies tremor in the fly model of SCA3.

A previous study in mice showed that ablation of GABAergic (Gad2-expressing) spinal interneurons that pre-synaptically inhibit sensory neurons led to tremor-like movements (Fink et al., 2014). Although we did not have a genetic entry point to manipulate neurons pre-synaptic to sensory neurons in flies, some of the interneurons post-synaptic to sensory proprioceptive neurons are GABAergic (Agrawal et al., 2020). We decided to test the DBD lines that underlie these interneuron populations (VT045623-DBD, VT044946-DBD and VT000606-DBD) by crossing them to R21G03-AD, as we were curious whether we could further narrow down a tremor-relevant subpopulation. MutATXN3-(CAG)_84_ driven by the intersection of R21G03-AD and VT045623-DBD led to significant tremor rates (Fig. 3C). Examination of this Split-Gal4 driver showed that it was expressed in a small population of ∼25-30 neurons in the 3- to 5-day-old adult VNC (Fig. 3D).

We analysed the neurotransmitter identities in this population of interest by immunostaining for anti-GABA (GABAergic neurons; *n*=7), anti-Vglut (also known as VGlut1) (glutamatergic neurons; *n*=5), anti-serotonin (serotonergic neurons; *n*=5) and anti-tyrosine hydroxylase (dopaminergic neurons; *n*=7). We found that ∼80% of these neurons were GABA positive (Fig. 3E; *n*=7). Therefore, dysfunction of a small population of ∼30 predominantly GABAergic neurons in the fly VNC is sufficient to cause leg tremors during walking.

### Morphological analysis of tremor-associated neurons and their post-synaptic partners

Next, we used the stochastic trans-Tango method of trans-synaptic mapping (Talay et al., 2017) to characterise post-synaptic partners of these ∼30 neurons. Using this method, Gal4-expressing neurons at the intersection of R21G03-AD+VT045623-DBD will be labelled green, while cells that are post-synaptic to these neurons will be labelled red, after heat shock, in a stochastic and mosaic manner, which allows the arborisation of individual neurons to be examined. Whereas flies that were not heat shocked showed no RFP expression in the VNC (Fig. S3A), heat-shocked flies showed RFP expression (*n*=18) (Fig. S3B). The post-synaptic neurons for the 18 flies exhibited several different types of morphologies (Fig. S3Bi-v). GABAergic input to Vglut-expressing leg motoneurons was previously shown to be required for normal walking; however, tremor was not investigated (Gowda et al., 2018). We hence carried out Vglut immunostaining and found that none of the post-synaptic neurons in the Trans-Tango experiment expressed Vglut. This was confirmed by low Manders' coefficients of colocalisation, suggesting that the neurons of interest are not interneurons pre-synaptic to motor neurons.

In summary, our results show that SCA3-induced tremor in the fly results from loss of GABAergic neuron function in the VNC. Hypoactivation of GABAergic neurons, but not of the other neurotransmitter populations tested, caused tremor. Loss of function of a small population of ∼30 neurons (R21G03-AD+VT045623-DBD), of which ∼80% are GABAergic, was sufficient to cause tremor. This driver provides a genetic entry point to characterise the circuitry and function of a group of neurons required for smooth motor function in the fly.

## DISCUSSION

Tremor is a common symptom of neurodegenerative diseases such as spinocerebellar ataxias. Animal and human studies have revealed the broad brain regions responsible for tremor (Miskin and Carvalho, 2018; Kuo et al., 2019). However, the specific neural circuitry dysfunctions that lead to tremor are still not well understood. In this study, we investigated the neuronal populations required for smooth motor control during walking using the genetically amenable *Drosophila* model. To the best of our knowledge, this is the first study in which a broad screen to identify neurons for which loss of function leads to tremor has been carried out, in any animal model. Such a study was previously limited owing to the high accuracy and throughput required to track and trace tremors. We were aided by FLLIT, a machine vision-based machine-learning method for leg tracking and tremor measurement (Wu et al., 2019). Using FLLIT, we screened and quantified tremors in different subsets of neurons and discovered that dysfunction of GABAergic neurons, and not that of other neurotransmitter populations tested, underlies tremor in the fly model of SCA3.

Having observed tremor in flies with pan-neuronal expression of mutATXN3-(CAG)_84_, but not alpha-synuclein [despite both showing climbing defects (Wu et al., 2019)], we first wanted to ask whether other poly-glutamine-associated transgenes could elicit tremor. Surprisingly, none of the tested lines showed tremor (Fig. S1A), whereas those with expression of Hid and Kir2.1 did (Fig. 1G). The phenocopy of the tremor phenotype by expression of tools for neuronal silencing and ablation, in the same neurons for which expression of mutATXN3 causes tremor, demonstrates that tremor will result if the function of this population of neurons is sufficiently inhibited, whether from the molecular perturbations caused by mutATXN3 protein or via other mechanisms. This mutATXN3-(CAG)_84_ fly model was generated by random P-element insertion (Warrick et al., 2005), and we previously found by inverse PCR that the transgene was inserted into the gene *effete/UbcD1*, which encodes a highly conserved E2 ubiquitin-conjugating enzyme (Treier et al., 1992). Although we did not determine whether the insertion site had an effect on *effete* expression or function, loss of function alleles of *effete* was previously found to exacerbate Atx1-induced neurodegeneration (Fernandez-Funez et al., 2000). Hence, the transgene insertion position may have sensitised our screen, allowing the identification of neuronal populations that are functionally important for smooth motor control using this transgene, but not other transgenes for expanded polyglutamine repeat proteins. Nonetheless, the transgene insertion did not have an effect on the majority of drivers screened, as most driver lines did not cause tremor when crossed to mutATXN3 (Fig. 1C; Table S1).

To cast the net widely, we first undertook a genetic screen using drivers expressed in broad populations of neurons. Successively intersecting the identified driver line with other drivers using genetic methods allowed us to narrow down the relevant population of neurons to ∼30 neurons (Fig. 3D) and ascertain that they are primarily GABAergic (Fig. 3D,E). This driver line can provide a genetic entry point to explore a circuitry that controls smooth locomotion in the fly model.

The fly VNC, like its homologous counterpart the vertebrate spinal cord, plays a critical role in processing and coordinating higher brain information for regulating locomotor activities. Although brain signals control complex behaviours (Braun et al., 2024), headless flies can walk (Yellman et al., 1997), indicating that higher-order brain regions contribute minimally to leg movement coordination, and that the VNC contains the fundamental circuitry necessary for generating walking behaviour. This aligns with our finding that dysfunction of the VNC population of the R21G03-Gal4 driver was necessary and sufficient to cause tremor.

Dysfunctions that occur during development can contribute to neurodegeneration. For example, increase in levels of Atxn1 throughout development due to loss of Pumilio causes very early-onset neurodegeneration in mice (Gennarino et al., 2015). These effects can be difficult to untangle from loss of function in the adult motor circuit and have important implications for how early therapeutic interventions need to occur. We found that dysfunction of a tremor-associated neuronal population only in adult stages is sufficient to induce tremor (Fig. 2B), demonstrating that these neurons function in adult circuitry to regulate smooth locomotion.

Functional studies have implicated dysregulation of GPCRs, components of the chaperone complex and Ca2^+^ transport and signalling, during SCA3 pathogenesis (Eidhof et al., 2019; McLoughlin et al., 2020; Zeng et al., 2018). Some of the proteins that function in these processes are being studied as potential therapeutic targets (Chen et al., 2020). Our results from transcriptomic analysis in the fly model showed a conservation of these pathway perturbations (Fig. S2H-J). Hence, the fly model recapitulates hallmarks of tremor and SCA3 pathogenesis at the molecular level, highlighting the usefulness of combining fly genetics with FLLIT and tremor quantitation to study tremor pathology.

Consistent with our previous findings in *Hyperkinetic* mutants of the beta subunit of the *Shaker* voltage-gated potassium (Kv) channel (Trout and Kaplan, 1973), which showed ∼60% tremor penetrance under similar recording parameters (Wu et al., 2019), tremor in *Drosophila* appears to be a specific behaviour with incomplete penetrance. This possibly arises from the stochastic combination of neurons affected with age within a population (Fig. S2F) and would be interesting to explore in a future study. The phenotypic penetrance, ranging from ∼25% to ∼80%, can be distinguished from the ∼1.2% background tremor rate, by using sufficiently large sample sizes and statistical analyses with multiple comparisons.

A key finding of our study is that dysfunction of a small population of GABAergic neurons is sufficient to cause tremor in a *Drosophila* model of SCA3. Of the tested neurotransmitter subtypes within the R21G03 population [of which the VNC component is necessary and sufficient to cause tremor (Fig. 1E,F)], only the loss of the GABAergic segment caused tremor (Fig. 3A). In addition, of the neurotransmitter Gal4-drivers used to drive mutATXN3-(CAG)_84_ across the nervous system, only Gad1-Gal4, which is expressed specifically in GABAergic neurons, led to a tremor phenotype (Fig. 3B). Given the fact that ∼38% of VNC neurons (∼10,000 neurons) are GABAergic (Allen et al., 2020), it is striking that dysfunction of only 30 neurons is sufficient to cause tremor.

ET is a prevalent movement disorder for which the GABA hypothesis is a prominent proposed mechanism. The primary sites of dysfunction in ET are thought to be the inferior olive in the brainstem (Park et al., 2010) and the cerebellum (Louis et al., 2014) (although other brain regions have been implicated). The GABA hypothesis posits that a disturbance in GABAergic neurotransmission in the cerebellum drives pathogenesis and progression of ET (Paris-Robidas et al., 2012; Bellows and Jimenez-Shahed, 2022; Gironell, 2022). The primary evidence supporting this hypothesis is that the majority of treatments for ET are known to modulate GABAergic signalling (Gironell, 2022). In addition, changes in concentrations of GABA receptors have been observed in the dentate nucleus of the cerebellum (Paris-Robidas et al., 2012). Hence, the GABA hypothesis in ET revolves around GABAergic signalling in the central brain and brainstem. However, the tremor-associated GABAergic neurons that we identified are located in the VNC and not the central brain. In mice, loss of pre-synaptic inhibition of sensory terminals by spinal GABAergic interneurons leads to forelimb tremor-like oscillations (Fink et al., 2014). Interestingly, a recent study in flies (Dallmann et al., 2025) provides evidence that GABAergic 9A interneurons suppress activity in movement-encoding hook proprioceptor axons during active leg movements via pre-synaptic inhibition, which is reminiscent of the above phenomenon in mice (Fink et al., 2014). We speculate that the population of ∼30 VNC GABAergic neurons we identified, the dysfunction of which results in tremor (Fig. 3D; Fig. S3B), comprise the GABAergic 9A interneurons identified by Dallmann and colleagues, and are functionally analogous to the mouse spinal interneurons identified by Fink and colleagues (Fink et al., 2014). Further investigation will be required to determine whether there is a conserved mechanism of smooth motor control from fly to mouse for which dysfunction can lead to tremors. Furthermore, this provides an alternative explanation for the observation that most ET drugs used in human patients modulate GABAergic signalling, and may suggest new drug targets that are specific to the spinal cord.

We still have no clear explanation for our observation that blocking of depolarisation of R21G03-Gal4 neurons using Kir2.1, but not their synaptic vesicle release by *shibire^ts^*, leads to tremor. If the GABAergic neurons of interest function through pre-synaptic inhibition of sensory axons via axo-axonal neurotransmission, it would be interesting to find out whether they are coupled through axo-axonal gap junctions, previously identified in hippocampal neurons (Schmitz et al., 2001), which could help to explain this observation. As tonic inhibition by activation of extrasynaptic GABA receptors can be generated in the absence of synaptic action potentials, e.g. from spillover of GABA from synapses, another potential explanation could be loss of tonic inhibition by extrasynaptic GABA receptors. These would be interesting avenues for future study.

### Limitations of the study

In this study, we systematically identified and validated a small subset of neurons in the fly VNC for which dysfunction underlies tremor using an automated machine-learning approach. Variability in the penetrance of the phenotype, which may point to differential rate of degeneration with age, or selective vulnerability to mutATXN3 aggregation within the affected neuronal population (Fig. S2F), merits future study. For example, single-cell RNA sequencing of the specific subset of GABAergic neurons (∼30) implicated in tremor generation could provide insights into transcriptomic alterations associated with the presence or absence of mutATXN3 accumulation, and signatures of cell degeneration or death, potentially elucidating the mechanisms that underlie phenotype variability. Additionally, mapping the pre- and post-synaptic partners of these tremor-associated neurons using recently available VNC connectomics atlases (Azevedo et al., 2024; Takemura et al., 2024) would be an important next step toward understanding, reconstructing and experimentally interrogating the neural circuitry responsible for this phenotype. Although these approaches are technically challenging and beyond the scope of the current study, they would substantially enhance our mechanistic understanding of the circuitry and mechanisms that underlie tremor.

We would also like to caveat that this study was limited to males owing to the technical and cost limitations of using both sexes. Although sex-specific behavioural differences (Asahina, 2018), including locomotor activity (Soyam and Kannan, 2024), have been well described in *Drosophila*, we note that an earlier report showed no significant differences in toxicity of UAS-mutATX3-Q80 in male versus female *Drosophila* (Blount et al., 2023). In human SCA3 cohorts, some reports have shown faster progression of non-ataxia symptoms in females; however, sex-associated clinical differences are largely not frequent in polyQ diseases, including SCA3 (Costa and Maciel, 2022). Consistent with this, knock-in mouse models of SCA3 also did not show sex-specific differences in aggregate formation (Haas et al., 2022). Based on these observations, we hypothesise that the role of tremor neurons identified in this study also extends to female *Drosophila*, although there may be slightly differing effects of aggregate formation in sexually dimorphic motor behaviours modulated by GABA, including courtship, copulation and aggression (Sengupta and Kravitz, 2025).

## MATERIALS AND METHODS

### Fly husbandry

The fly lines used in this study are listed in Table S7. Unless otherwise stated, all fly stocks and crosses were reared on standard yeast-cornmeal-agar medium at 25±1°C, 70% humidity in an environmentally controlled incubator on a 12-h light-dark schedule. For some experiments crosses, and progeny were maintained at 28±1°C. All the crosses and the progeny of experiments using RNAi lines were maintained at 28°C. Owing to the technical and cost limitations of using both sexes, only male flies were used in experiments. Please see Discussion for potential differences that may be observed if female flies were used in the study.

The experiment in which TrpA1 and Kir2.1 were compared with *shibire^ts^* was carried out at 28°C, as TrpA1 activity is temperature dependent. Flies to be compared were kept at the same temperature in a given experiment.

Although wing defects were previously observed by pan-neuronal expression of a truncated form of mutATXN3 (UAS-MJDtrQ78) (Chakravorty et al., 2022), which appears to cause more severe phenotypic effects in general (Warrick et al., 2005, 1998), we did not observe wing defects in Elav-GAL4>UAS-mutATXN3-(CAG)_84_ flies.

### Climbing assays

Climbing assays were performed as described in Wu et al. (2019). Briefly, the maximum climbing height reached by single flies in 14 ml tubes cut at both ends (352059, Falcon) within 30 s was measured and averaged over two technical replicates. For the initial GAL4 screen, single fly climbing assays were carried out at 50±1 days, and the three poorest climbers were recorded for FLLIT analysis.

Based on the initial Gal4 screen, we aimed to carry out climbing assays and selection of the poorest climbers for FLLIT recording and tremor analysis at 50±1 days post-eclosion. However, for experiments involving genetic drivers (Elav-Gal4, Ple-Gal4, ChAT-Gal4) or transgenic lines (UAS-Kir2.1, UAS-Hid), premature lethality was observed, preventing assessment at 50 days. Hence, climbing ability was monitored over time, and flies were analysed for tremor at the age at which ∼50% of the flies were unable to climb to the top of the assay tube. Thus, in some cases, climbing assays to determine tremor analysis were performed at an earlier age. For instance, single-fly climbing assays involving progeny from Elav-Gal4 crosses were conducted at 22±1 days. In the Split-Gal4 screen (Fig. 2A), climbing assays were performed at 57±1 days.

### Fly gait recording and processing using FLLIT

Flies were recorded and analysed for tremor after climbing assays were carried out (see ‘Climbing assays’ section). Based on the climbing assay results, all flies that climbed between ∼0.3 and 0.9 cm were recorded for gait analysis All fly recordings were processed by FLLIT, as previously described (Banerjee et al., 2020; Wu et al., 2019). Briefly, a Photron FastCam MC2 high-speed camera was positioned below the sample arena, which was backlit with a diffused infrared LED array. Flies were placed in the arena using mouth aspiration or briefly cooled on ice, and allowed to acclimatise for at least 5 min (mouth aspiration) or 15 min (on ice) before recording. The arena's floor and ceiling were glass microscope slides, with 1.5-mm thick transparent acrylic walls. To ensure consistency, the first two initial instances of straight walking behaviour across the arena without touching the wall were recorded. The field of view was 10×10 mm. Videos were recorded at 1000 frames/s with 512×512 resolution at 25°C. Flies were recorded walking in a straight line for at least 1.5 body lengths in distance. Videos were then processed and tracked using FLLIT.

### Tremor analysis

After leg tracking was completed, the first video recorded was analysed. The body-centred leg claw positions were further processed in MATLAB (MathWorks) (Wu et al., 2019) (https://github.com/Clibedinsky/FLITT_tremor_analyses.git), where shaking and tremor events were analysed as described (Wu et al., 2019). Briefly, to quantify the number of shaking movements, local extrema of at least 3 pixels in amplitude after normalisation to body size were identified from the traces (black or red circles, Fig. 1B). A cutoff of 3 pixels was chosen to filter out small displacements that occur owing to tracking errors (because ∼98% of tracked positions were within 3 pixels of the actual leg claw as located by manual annotation and roughly corresponded to the width of a leg tip) (Wu et al., 2019). As tremors are periodic shaking movements, we then looked for tremor-like episodes during walking: a series of three or more shaking events separated by <100 ms [conservatively chosen based on the average stride rate of ∼10 Hz in control flies (Wu et al., 2019)]. Each of these events was defined as a tremor event (red circles, Fig. 1B).

### Immunohistochemistry

Brains (central brain and/or VNC) of 8-day-old flies (or as specifically stated for each experiment) were dissected in ice-cold 1× PBS and fixed in 4% paraformaldehyde (PFA; 15710, Electron Microscopy Sciences) for 20 min at room temperature, then washed for 3×10 min in 1× PBS with 0.1% Triton X-100 (PBT), followed by blocking with 3% bovine serum albumin (BSA) for 16-20 h at 4°C. Samples were then incubated in primary antibody for 36-48 h at 4°C. Antibodies used were as follows: chicken anti-GFP (1:1000, ab13970, Abcam), rabbit anti-Myc (1:500, 71D10, Cell Signaling Technology), rabbit monoclonal antibody (2278, Cell Signaling Technology), rabbit anti-Mcd8-RFP (1:500, 632496, Clontech), mouse anti-Nc82 (1:100, Developmental Studies Hybridoma Bank), rabbit anti-GABA (1:1000, A2052, Sigma-Aldrich), rat anti-HA (1:200, 11867423001, clone 3F10, Roche), rabbit anti-TH (1:100, 22941, Immunostar), rabbit anti-serotonin (1:1000, S5545, Sigma-Aldrich) and rabbit anti-Vglut [1:10000, gift from Aaron DiAntonio (Daniels et al., 2008)]. Samples were washed 4×15 min in PBT and incubated with secondary antibodies anti-chicken AF488, anti-rabbit AF555 or anti-mouse AF633 (Invitrogen) for 16-20 h at 4°C depending on the experiment, washed 4×10 min in PBT and stained with DAPI (1:3000, Thermo Fisher Scientific) for 20 min before mounting in VectaShield (Vector Laboratories). All fluorescence images were obtained on a Zeiss LSM 700 confocal microscope at 20× or 40× magnification at 1 µm or 1.9 µm intervals. The images were processed using Zen 2012 (Zeiss) or ImageJ [National Institutes of Health (NIH), Public Domain, BSD-2] and Photoshop (Adobe). For visualising the potential intersectional expression pattern of the Split-Gal4 AD and DBD lines, virgins of 70473-AD; UAS-CD4-tdGFP were crossed with each of the potentially overlapping DBD males. Late third-instar larval brains and VNCs were dissected in chilled PBS and fixed in 4% PFA for 20 min. After washing for 3×10 min with 0.1% PBT, the brains and VNCs were incubated with DAPI. The mounted brains and VNCs were imaged under a Zeiss LSM 700 confocal microscope at 20× magnification. Cell counting and colocalisation analyses were carried out using ImageJ (FIJI) and Imaris software. Details on the specific analyses are described in the sections below.

### Cell counting

The whole brains and VNCs of 8-day-old flies were dissected, fixed and immunostained as described above. All brains and VNCs were mounted ventral up and scanned with *z*-optical sections of 1 µm interval. For counting UAS-Histone-RFP cells, samples were scanned at 20× and 40. In both cases, the images were first converted to 16 bit followed by maximum projection using ImageJ. After extracting the red channel, the threshold was adjusted and watershade separation was done. The numbers of Histone-RFP cells were then counted using the Analyze Particle function in ImageJ. For 20× whole-brain images, the numbers of cells in central brain and VNC were counted by selecting the region of interest. To count Histone-RFP cells specifically in T1 and T2 VNC segments of Split-Gal4 flies, they were filtered out with reference to the DIC channel and counted as outlined above.

### Colocalisation analysis

Colocalisation quantitative analysis between anti-GABA and R21G03-AD+VT045623-DBD>UAS-Histone-RFP+UAS-tdGFP (Fig. 3E) was carried out using Imaris 9.9. ‘Spots object creator’ was used for Histone-RFP; ‘Surface object creator’ was used for anti-GFP and anti-GABA. Background subtraction and manual thresholding were applied for both spot and surface objects. The filter ‘Shortest Distance between 2 objects below 0.1 µm’ was applied to identify the colocalisation between each channel.

Colocalisation analysis between GABA (green) and Vglut (blue) (Fig. S3B) was performed using the Imaris Coloc module to quantitatively assess and generate the scatter plot using the spatial overlap between the green (GABA) and Vglut fluorescence channels. A constant region of interest shown as the upper-right quadrant was selected from the scatter plot for all the images to calculate the Manders’ colocalisation coefficients for both channels.

### Single-cell dissociation

The VNC dissociation protocol was adapted from Croset et al. (2018). Briefly, for each of our experiments, 60-70 VNCs from 7- to 9-day-old flies were individually dissected in ice-cold calcium- and magnesium-free DPBS (Gibco, 14190-086) and immediately transferred into 1 ml toxin-supplemented Schneider's medium (tSM; Schneider's medium, Gibco, 21720-001+50 µM d(−)−2-amino-5-phosphonovaleric acid, 20 µM 6,7-dinitro quinoxaline-2,3-dione and 0.1 µM tetrodotoxin) on ice. VNCs were washed once with 1 ml tSM and incubated in tSM containing 1.11 mg/ml papain (Sigma-Aldrich, P4762) and 1.11 mg/ml collagenase I (Sigma-Aldrich, C2674). The dissected VNCs were washed once more with tSM and subsequently triturated with flame-rounded 200 µl pipette tips. Dissociated VNCs were resuspended into 600 μl PBS+0.01% BSA and filtered through a 10 µm CellTrix strainer (Sysmex, 04-0042-2314).

### RNA sequencing

Dissociated cell suspensions were sorted by FACS. Live (DAPI^−^) GFP^+^ or GFP^−^ cells were sorted directly into PCR tubes at 500 cells per replicate tube, containing 2 µl of 1 mg/ml BSA (Ambion) with 4 U/μl Recombinant RNase Inhibitor (Takara Bio) and 1 µl of 10 mM dNTP mix, then snap frozen and stored at −80°C. cDNA libraries were prepared using the Smart-seq2 protocol (Picelli et al., 2014) with the following modifications: (1) change in lysis buffer and cell collection as described; (2) addition of 20 μM template switching oligonucleotide; (3) 21 PCR cycles for cDNA amplification; (4) use of 200 pg cDNA with 1/5 reaction of Nextera XT kit (Illumina). The length distributions of the cDNA and Nextera libraries were monitored using a DNA High Sensitivity Reagent Kit on the Perkin Elmer Labchip. All samples passing QC were sequenced on an Illumina HiSeq X system targeting 15 million reads/sample at 2×151 cycles.

### Kir2.1-FLP heat-shock time-course experiment

Flies were raised in a 25°C incubator on standard cornmeal-agar medium. After removal of parents, progeny collected in plastic food vials were subjected to heat shock in a 37°C water bath for 1 h at embryo, pupal or adult stages. For the adult heat-shock condition, flies were sorted 1 day after eclosion before undergoing heat shock. For the other stages/conditions, the desired genotypes were sorted and collected after eclosion. Progeny of the correct genotype [hsFLP (X)/Y; FRT.stop. UAS-Kir2.1, R21G03-AD; R47B12-DBD] were aged between 25 and 30 days. The poorest climbers from each condition were selected and recorded for tremor analysis.

### Multi-colour FLPout

We stochastically labelled the individual neurons using multicolour flip out technique as previously described (Nern et al., 2015). hsFLP; R21G03-DBD/TM3 virgins were crossed with R21G03-AD wtATXN3; UAS≫HA UAS≫V5 UAS≫FLAG/TM3 males, and hsFLP; +; R21G03-DBD/TM3 females were crossed with R21G03-AD; UAS≫HA UAS≫V5 UAS≫FLAG mutATXN3/TM2 males. For induction of Flp recombinase, 7±1-day-old progeny males were heat shocked by immersing the vials in a 37°C water bath for 12 min (sparse labelling). The heat-shocked flies were maintained at 25°C and dissected after 2 days followed by immunostaining as described above.

### Trans-Tango

UAS-myrGFP, QUAS-mtdTomato (3xHA), hsFLP (X); trans-Tango, FRTG13, tubP-QS(5A)/R21G03-AD (II); VT045623-DBD/+ (III) were raised at 18°C and transiently heat shocked in a 37°C water bath for 1 h during third-instar larval stage.

### Quantification and statistical analysis

All experiments were carried out at least twice, with consistent results. We used standard sample sizes, from other related studies, and similar to previous work. Exact sample sizes are indicated in the figures and/or figure legends. Data were analyzed using Prism (GraphPad Software). All data were presented as mean±s.e.m. For comparing the means of two groups, a non-parametric Mann–Whitney test was used. For analysis of multiple groups, one-way ANOVA was utilised. *P*<0.05 was considered statistically significant.

## Supplementary Material

10.1242/dmm.052329_sup1 Supplementary information

Table S1. List of fly genotypes of GAl4 drivers used to express mutATXN3-(CAG)_84_ for initial tremor screen.

Table S2. Split-GAL4 screen to investigate DBD lines that intersect with R21G03-AD, using a UAS-tdGFP reporter.

Table S3. RNAseq analysis of FACS-sorted VNC neurons from nsyb-Gal4 > UAS-GFP flies.

Table S4. RNA-seq analysis of FACS-sorted VNC neurons from R21G03-Gal4 > UAS-tdGFP flies.

Table S5. RNA-seq analysis identifying differentially expressed genes in FACS-sorted VNC neurons of R21G03-Gal4 > UAS-tdGFP flies compared to nsybGal4 > UAS-tdGFP flies.

Table S6. RNA-seq analysis listing genes differentially expressed when mutATXN3 was expressed in tremor neurons compared to expresion of wild-type ATXN3.

Table S7. List of fly stocks used in this study
