# Supplementary material for: GABAergic neuronal dysfunction underlies tremor in spinocerebellar ataxia 3
Source: Dis Model Mech. 2025 Oct 29;18(10):dmm052329. doi: 10.1242/dmm.052329 (PMC12590474; doi:10.1242/dmm.052329)
Supplement: Supplementary information [file dmm-18-052329-s1.pdf]

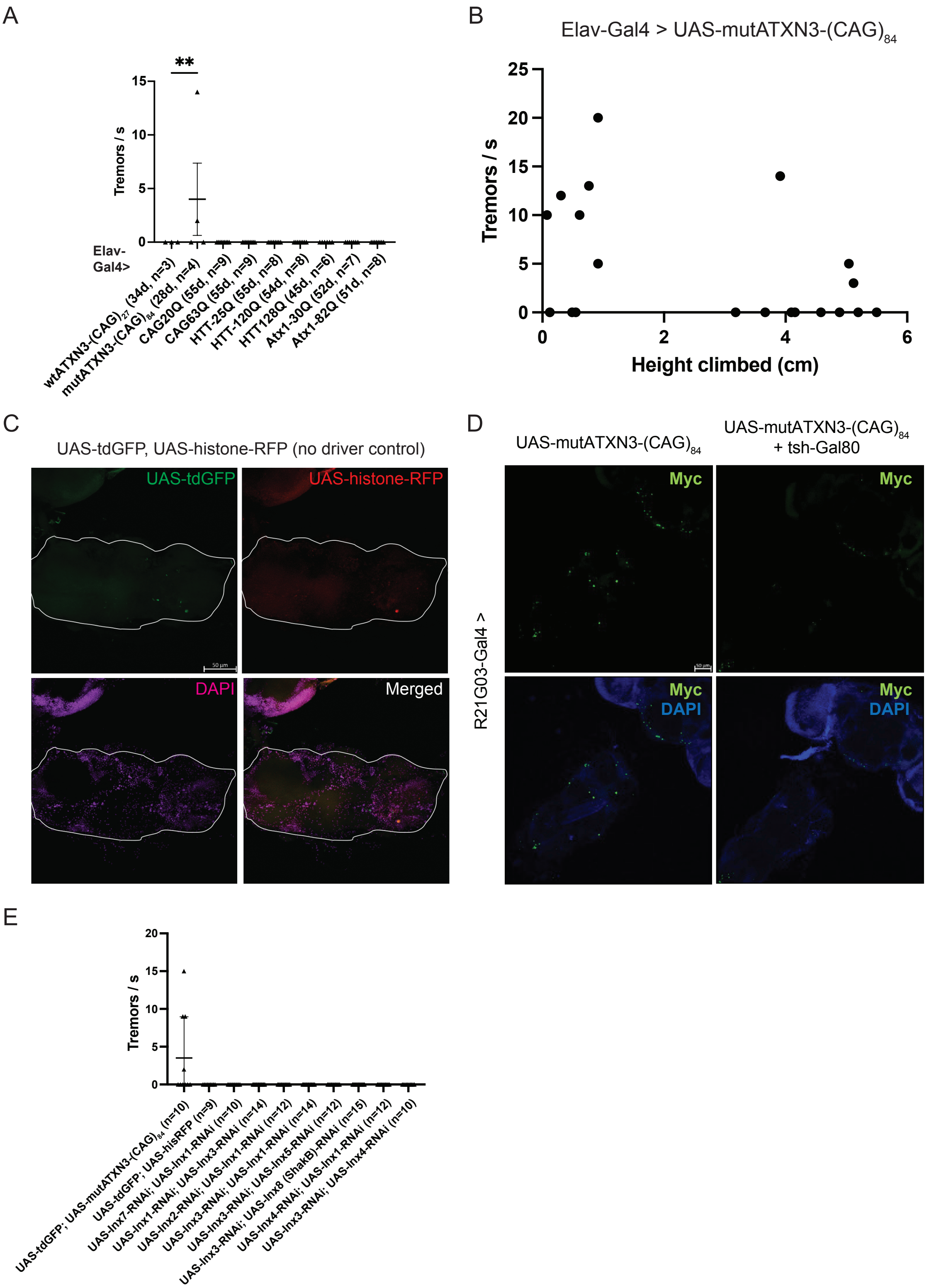
A
**
Tremors / s
Elav-Gal4>
wtATXN3-(CAG)$_{27}$ (34d, n=3)
mutATXN3-(CAG)$_{84}$ (28d, n=4)
CAG20Q (55d, n=9)
CAG63Q (55d, n=9)
HTT-25Q (55d, n=8)
HTT-120Q (54d, n=8)
HTT128Q (45d, n=6)
Atx1-30Q (52d, n=7)
Atx1-82Q (51d, n=8)
B
Elav-Gal4 > UAS-mutATXN3-(CAG)$_{84}$
Tremors / s
Height climbed (cm)
C
UAS-tdGFP, UAS-histone-RFP (no driver control)
UAS-tdGFP
UAS-histone-RFP
DAPI
Merged
D
UAS-mutATXN3-(CAG)$_{84}$
UAS-mutATXN3-(CAG)$_{84}$ + tsh-Gal80
R21G03-Gal4 >
Myc
Myc
DAPI
E
Tremors / s
UAS-tdGFP; UAS-mutATXN3-(CAG)$_{84}$ (n=10)
UAS-tdGFP; UAS-hisRFP (n=9)
UAS-Inx7-RNAi; UAS-Inx1-RNAi (n=10)
UAS-Inx1-RNAi; UAS-Inx3-RNAi (n=14)
UAS-Inx2-RNAi; UAS-Inx1-RNAi (n=12)
UAS-Inx3-RNAi; UAS-Inx1-RNAi (n=14)
UAS-Inx3-RNAi; UAS-Inx5-RNAi (n=12)
UAS-Inx8 (ShakB)-RNAi (n=15)
UAS-Inx4-RNAi; UAS-Inx1-RNAi (n=12)
UAS-Inx3-RNAi; UAS-Inx4-RNAi (n=10)

Fig. S1. Refers to Figure 1

A. Pan-neuronal expression of poly-CAG (CAG20Q, n=9; CAG63Q, n=9), expanded ATXN1 (Atx-30Q, n=7; Atx1-82Q, n=8) and HTT (HTT-25Q, n=8; HTT-120Q, n=8; HTT120Q, n=6; Atx1-82Q, n=6) do not result in tremor (All non-significant by one-way ANOVA with Dunn's multiple correction; $^{**}p < 0.01$). Also tested: mutATXN3-$(CAG)_{27}$, n=3; mutATXN3-$(CAG)_{84}$, n=4.

B. Comparison of tremor rate vs height climbed for single Elav-Gal4-driven UAS-mutATXN3-$(CAG)_{84}$ flies shows that poorer climbers (n=9) tend to show greater rates of tremor compared to good climbers (n=10) (correlation coefficient (r) = -0.44; p = 0.0516), although some good climbers also exhibited tremor.

C. Undriven UAS-tdGFP; UAS-histone-RFP controls have minimal expression in the adult VNC. Scale bar: 50μm.

D. Blocking R21G03-Gal4 expression in the VNC using tsh-Gal80 diminished expression of mutATXN3-$(CAG)_{84}$-myc expressed from this driver in the VNC. Scale bar: 50μm.

E. RNAi-knockdown of innexins in pairs using R21G03-Gal4 did not elicit tremor. (Sample sizes as shown in the figure. All non-significant by one-way ANOVA with Dunn's multiple correction).

All experiments were carried out at least twice. Tremor analyses were carried out on single fly biological replicates. All statistics were carried out with non-parametric Mann-Whitney (when comparing two samples) and one-way ANOVA with Dunn's multiple correction (when comparing more than two samples). Bars show the means and error bars reflect standard error of the mean. $^{*}p < 0.05$; $^{**}p < 0.01$; $^{***}p < 0.001$; $^{****}p < 0.0001$

Disease Models & Mechanisms • Supplementary information

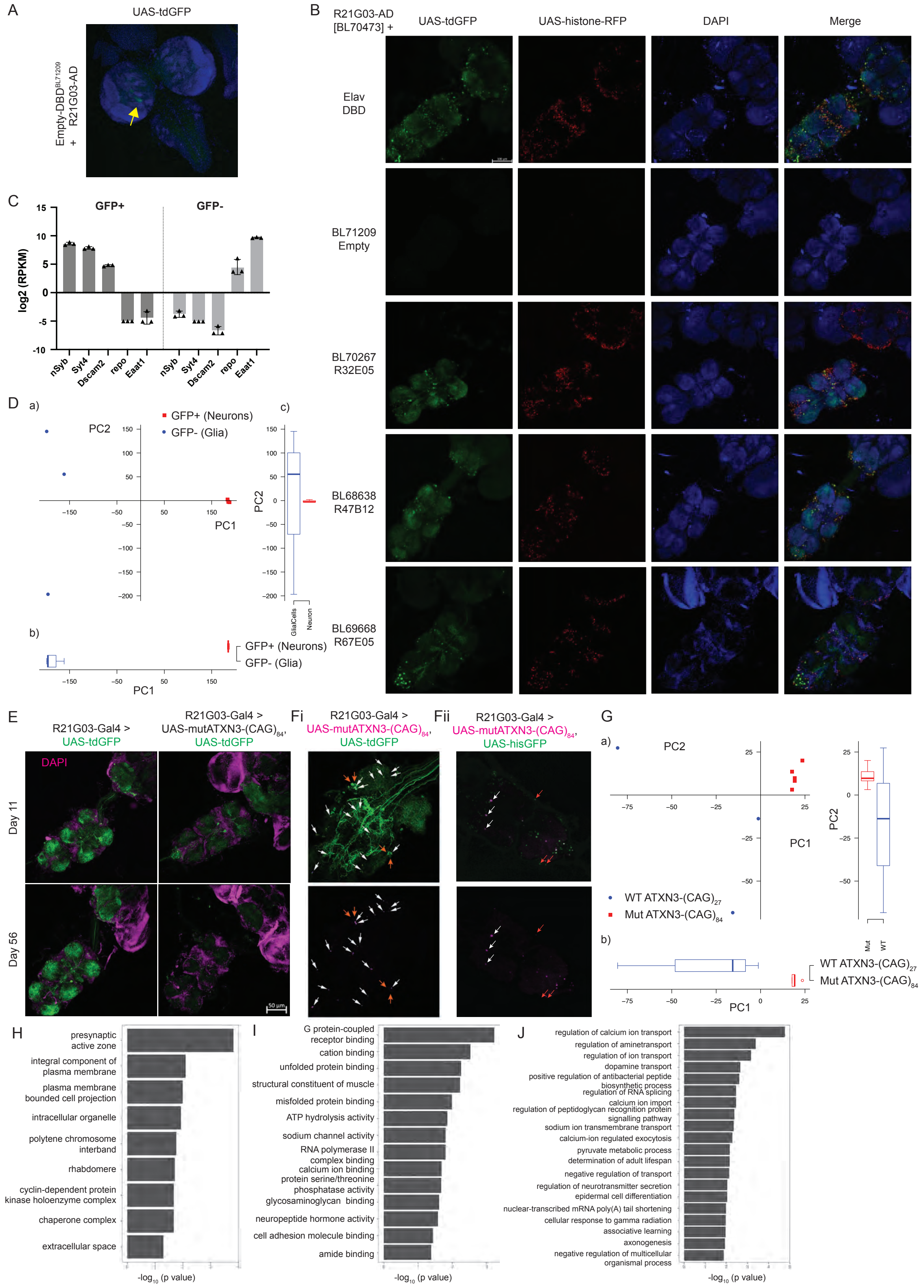

Disease Models & Mechanisms • Supplementary information

Fig. S2. Refers to Figure 2.

A. R21G03-AD + Empty-DBD shows expression in neuroblast-like cells in the larval central brain (yellow arrow).

B. Expression of R21G03-AD when intersected with Elav-DBD, Empty-DBD, R32E05-DBD, R47B12-DBD or R67E05-DBD. Scale bar: 100μm.

C. FACS-sorted nsyb-Gal4 > UAS-tdGFP cells showed upregulation of neuronal genes and down-regulation of glial genes compared to non-GFP cells from the same experiment (n=3).

D. Principal component (PC) analysis of the global gene expression patterns of GFP+ (neurons) and GFP- (glia) cells based on $\log_2$RPKM values. a) Scatter plot with PC1 and PC2 which account for 59% and 18% of the total variance of the data showing separation and clustering of types of cells and samples; boxplot of b) PC1 and c) PC2 stratified by type of cell.

E. Expression of mut-ATXN3-$(CAG)_{84}$ leads to reduced number of R21G03-GAL4-expressing neurons (labelled with UAS-tdGFP) with age, compared to control. Scale bar: 50μm.

Fi. Expression of UAS-mut-ATXN3-$(CAG)_{84}$-myc and UAS-tdGFP with R21G03-Gal4 reveals that while most tdGFP-expressing neurons also exhibit mutATXN3-$(CAG)_{84}$ aggregation (white arrows), some tdGFP-expressing neurons do not exhibit mutATXN3-$(CAG)_{84}$-myc aggregates (orange arrow), suggesting differential vulnerability to ATXN3 aggregation within the population.

Fii. Expression of UAS-mut-ATXN3-$(CAG)_{84}$-myc and UAS-histone-RFP (pseudo-coloured in green) with R21G03-Gal4 reveals that while some cells that express histone-RFP also exhibit mutATXN3-$(CAG)_{84}$ aggregates (in pink, white arrows), some cells that express histone-RFP do not exhibit mutATXN3-$(CAG)_{84}$ aggregation (orange arrows), supporting differential vulnerability to ATXN3 aggregation within the population. In this figure, the Mander's Coefficient for fraction of histone-RFP that overlaps with mutATXN3-$(CAG)_{84}$-myc is 0.366, and the Mander's Coefficient for fraction of mutATXN3-$(CAG)_{84}$-myc that overlaps with histone-RFP is 0.851. Therefore, mutATXN3 largely overlaps with histone-RFP signal, while only a small fraction of cells that express the driver show mutATXN3 aggregation.

Disease Models & Mechanisms • Supplementary information

G. Principal component (PC) analysis of the global gene expression profiles of wild type (wtATXN3-$(CAG)_{27}$) and mutant (mutATXN3-$(CAG)_{84}$) samples based on log_2RPKM values of all detectable genes. a) Scatter plot with PC1 and PC2, which respectively account for 25.3% and 18% of the total variance of the data showing separation and clustering samples; boxplot of b) PC1 and c) PC2 for the wild type and mutant.

H-J. Gene ontology (GO) enrichment analysis for genes differentially expressed between flies expressing wtATXN3-$(CAG)_{27}$ and mutATXN3-$(CAG)_{84}$, with p-value < 0.05. The barplots show the top enriched gene ontology (GO) terms of **G)** Cellular Component (CC), **H)** Molecular Function (MF) and **I)** Biological Process (BP). If there were more than 20 enriched terms, only the top 20 are shown (see Table S6 for the full list).

Disease Models & Mechanisms • Supplementary information

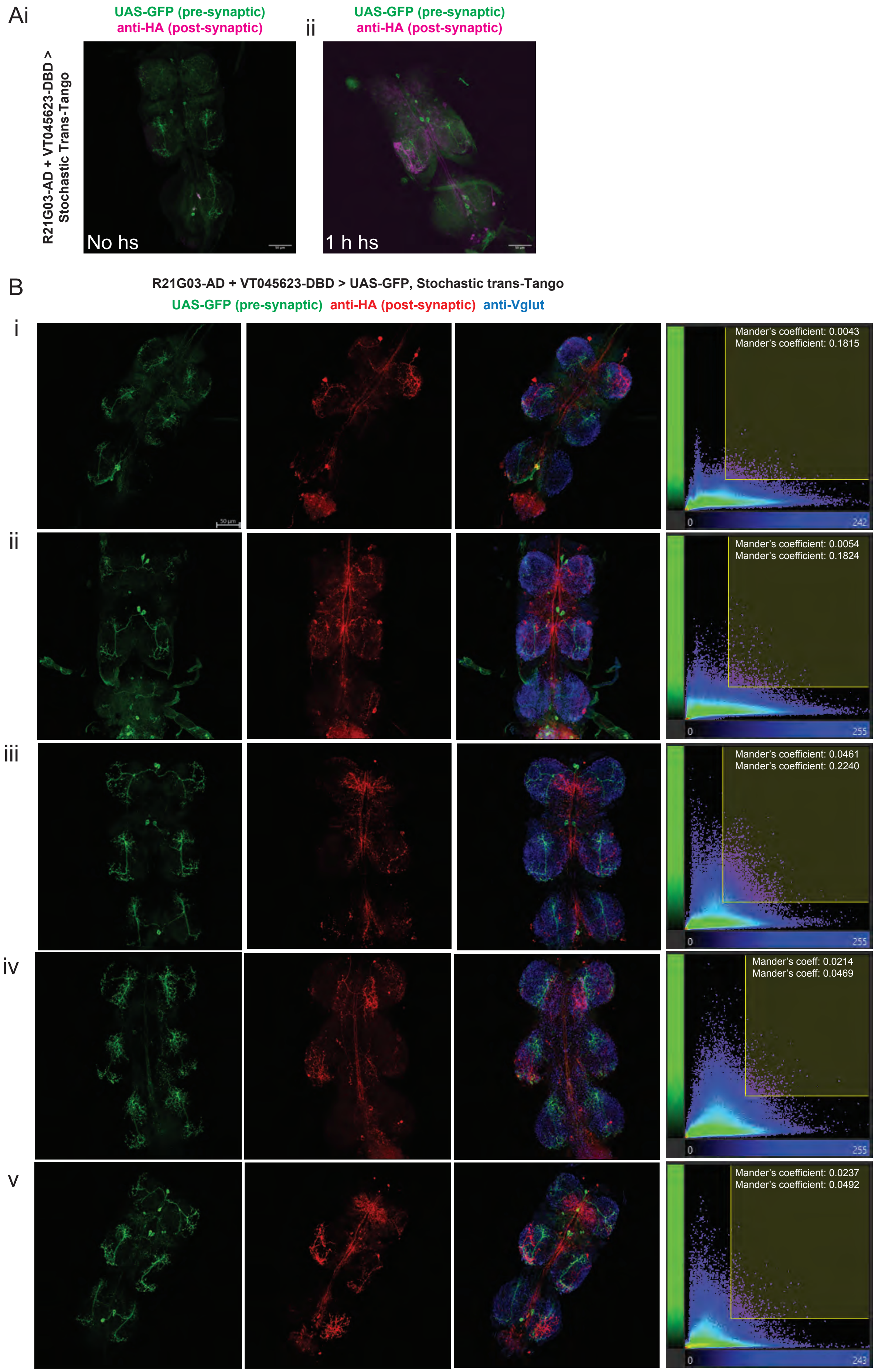

Ai
UAS-GFP (pre-synaptic)
anti-HA (post-synaptic)
ii
UAS-GFP (pre-synaptic)
anti-HA (post-synaptic)
R21G03-AD + VT045623-DBD > Stochastic Trans-Tango
No hs
1 h hs
B
R21G03-AD + VT045623-DBD > UAS-GFP, Stochastic trans-Tango
UAS-GFP (pre-synaptic) anti-HA (post-synaptic) anti-Vglut
i
Mander's coefficient: 0.0043
Mander's coefficient: 0.1815
0
242
ii
Mander's coefficient: 0.0054
Mander's coefficient: 0.1824
0
255
iii
Mander's coefficient: 0.0461
Mander's coefficient: 0.2240
0
255
iv
Mander's coeff: 0.0214
Mander's coeff: 0.0469
0
255
v
Mander's coefficient: 0.0237
Mander's coefficient: 0.0492
0
243

Fig. S3. Refers to Figure 3.

Trans-Tango experiments to examine neurons post-synaptic to tremor-associated neurons.

Ai. Trans-Tango flies that were not heat-shocked do not express HA. **ii.** Trans-Tango flies were heat-shocked express RFP. Scale bar: 50μm.

B. Examples of stochastic VT045623-DBD + R21G03-AD (green) Trans-Tango neurons, with their post-synaptic counterparts labelled in red. The scatter plot showing the extent of colocalization between the green (channel A) and Vglut (channel B) fluorescence signals. The Mander's coefficients mentioned in the inset was calculated based on the yellow box at the right quadrant representing the region where colocalization would be expected. The colour gradient depicts the density of pixels with corresponding intensity values with purple indicating low intensity and white indicating high intensity. Scale bar: 50μm.

Disease Models & Mechanisms • Supplementary information

Table S1. List of fly genotypes of GAl4 drivers used to express mutATXN3-(CAG)$_{84}$ for initial tremor screen. (A) GAL4 driver lines crossed with UAS-mutATXN3 and screened in initial primary screen for tremor. (B) GAL4 driver lines crossed with UAS-mutATXN3 and screened in second primary screen for tremor (defined as 3 shakes per second) - All flies did not show tremor.

Available for download at
https://journals.biologists.com/dmm/article-lookup/doi/10.1242/dmm.052329#supplementary-data

Table S2. Split-GAL4 screen to investigate DBD lines that intersect with R21G03-AD, using a UAS-tdGFP reporter.

Available for download at
https://journals.biologists.com/dmm/article-lookup/doi/10.1242/dmm.052329#supplementary-data

Table S3. RNAseq analysis of FACS-sorted VNC neurons from nsyb-Gal4 > UAS-GFP flies.

Available for download at
https://journals.biologists.com/dmm/article-lookup/doi/10.1242/dmm.052329#supplementary-data

Table S4. RNA-seq analysis of FACS-sorted VNC neurons from R21G03-Gal4 > UAS-tdGFP flies.

Available for download at
https://journals.biologists.com/dmm/article-lookup/doi/10.1242/dmm.052329#supplementary-data

Table S5. RNA-seq analysis identifying differentially expressed genes in FACS-sorted VNC neurons of R21G03-Gal4 > UAS-tdGFP flies compared to nsybGal4 > UAS-tdGFP flies.

Available for download at
https://journals.biologists.com/dmm/article-lookup/doi/10.1242/dmm.052329#supplementary-data

Table S6. RNA-seq analysis listing genes differentially expressed when mutATXN3 was expressed in tremor neurons compared to expresion of wild-type ATXN3.

Available for download at
https://journals.biologists.com/dmm/article-lookup/doi/10.1242/dmm.052329#supplementary-data

Table S7. List of fly stocks used in this study

Available for download at
https://journals.biologists.com/dmm/article-lookup/doi/10.1242/dmm.052329#supplementary-data

Disease Models & Mechanisms • Supplementary information

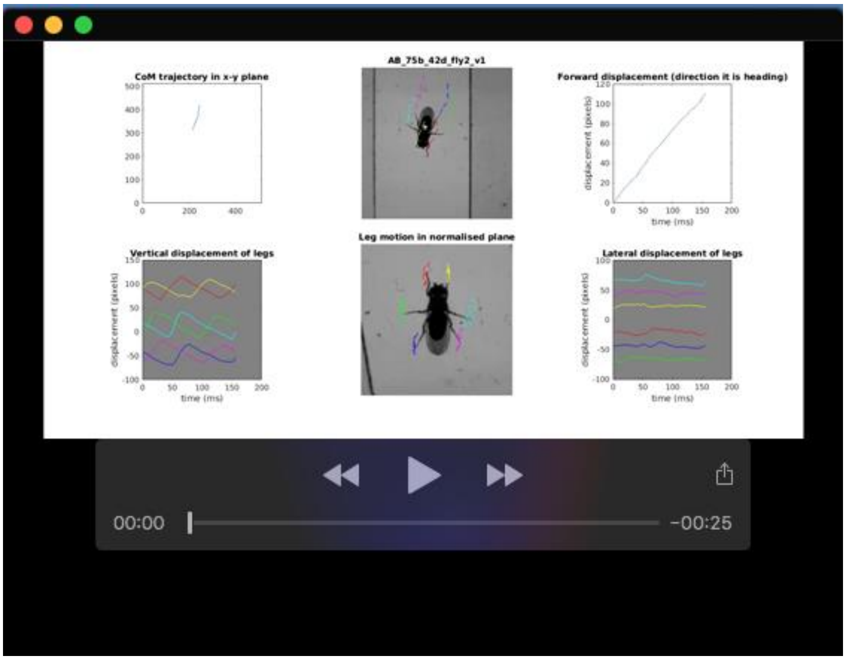

Movie 1. FLLIT-tracked video of a representative ElavGal4 > UAS-wtATXN3-$(CAG)_{27}$ fly.

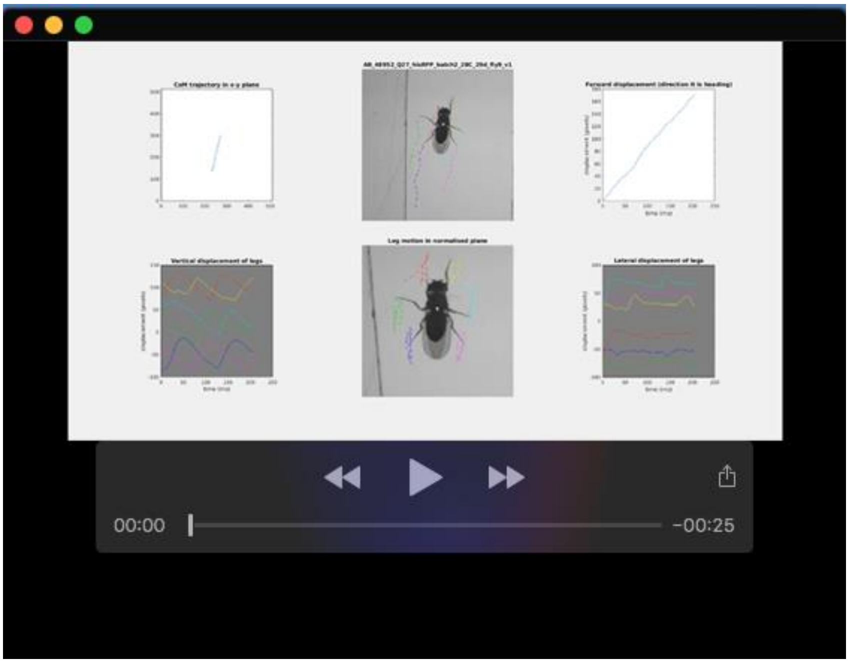

Movie 2. FLLIT-tracked video of a representative ElavGal4 > UAS-mutATXN3-$(CAG)_{84}$ fly.

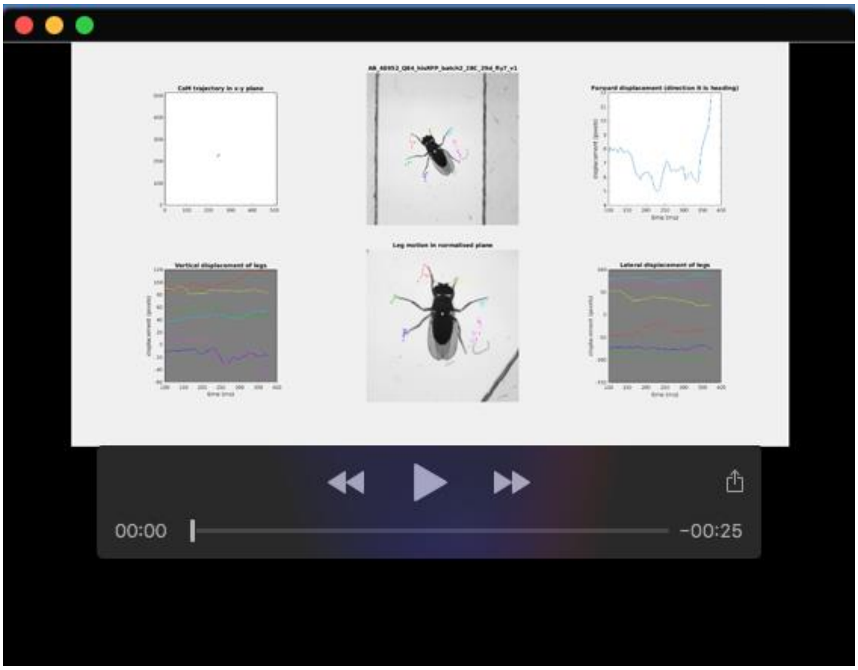

Movie 3. FLLIT-tracked video of a representative R21G03-Gal4 > UAS-wtATXN3-$(CAG)_{27}$-histone-RFP fly.

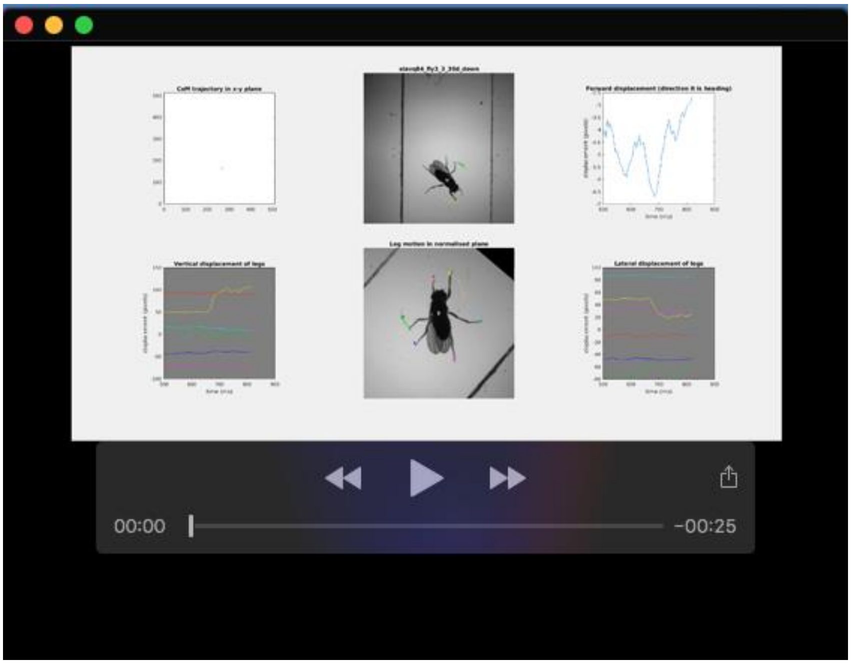

Movie 4. FLLIT-tracked video of a representative R21G03-Gal4 > UAS-mutATXN3-$(CAG)_{84}$-histone-RFP fly.
